# Repeated head trauma causes neuron loss and inflammation in young athletes

**DOI:** 10.1038/s41586-025-09534-6

**Published:** 2025-09-17

**Authors:** Morgane L. M. D. Butler, Nida Pervaiz, Kerry Breen, Samantha Calderazzo, Petra Ypsilantis, Yichen Wang, Julia Cammasola Breda, Sarah Mazzilli, Raymond Nicks, Elizabeth Spurlock, Marco M. Hefti, Kimberly L. Fiock, Bertrand R. Huber, Victor E. Alvarez, Thor D. Stein, Joshua D. Campbell, Ann C. McKee, Jonathan D. Cherry

**Affiliations:** 1 Department of Anatomy and Neurobiology, Boston University Chobanian and Avedisian School of Medicine, Boston, MA USA; 2 Boston University Alzheimer’s Disease and CTE Centers, Boston University Chobanian and Avedisian School of Medicine, Boston, MA USA; 3 Section of Computational Biomedicine, Department of Medicine, Boston University Chobanian and Avedisian School of Medicine, Boston, MA USA; 4 Department of Pharmacology, Physiology, and Biophysics, Boston University Chobanian and Avedisian School of Medicine, Boston, MA USA; 5 Department of Pathology and Laboratory Medicine, Boston University Chobanian and Avedisian School of Medicine, Boston, MA USA; 6 VA Boston Healthcare System, Jamaica Plain, MA USA; 7 VA Bedford Healthcare System, Bedford, MA USA; 8 Department of Pathology, University of Iowa Health Care, Iowa City, USA; 9 Iowa Neuropathology Resource Laboratory and Department of Pathology, University of Iowa, Iowa City, IA USA; 10 National Center for PTSD, VA Boston Healthcare System, Boston, MA USA; 11 Department of Neurology, Boston University Chobanian and Avedisian School of Medicine, Boston, MA USA

**Keywords:** Neurodegeneration, Neuroimmunology

## Abstract

Repetitive head impacts (RHIs) sustained from contact sports are the largest risk factor for chronic traumatic encephalopathy (CTE)^1–4^. Currently, CTE can only be diagnosed after death and the events that trigger initial hyperphosphorylated tau (p-tau) deposition remain unclear^2^. Furthermore, the symptoms endorsed by young individuals are not fully explained by the extent of p-tau deposition^2^, severely hampering therapeutic interventions. Here we observed a multicellular response prior to the onset of CTE p-tau pathology that correlates with number of years of RHI exposure in young people (less than 51 years of age) with RHI exposure, the majority of whom played American football. Leveraging single-nucleus RNA sequencing of tissue from 8 control individuals, 9 RHI-exposed individuals and 11 individuals with low-stage CTE, we identify SPP1-expressing inflammatory microglia, angiogenic and inflamed endothelial cells, astrocytosis and altered synaptic gene expression in those exposed to RHI. We also observe a significant loss of cortical sulcus layer 2/3 neurons independent of p-tau pathology. Finally, we identify TGFβ1 as a potential signal that mediates microglia–endothelial cell cross talk. These results provide robust evidence that multiple years of RHI is sufficient to induce lasting cellular alterations that may underlie p-tau deposition and help explain the early pathogenesis in young former contact sport athletes. Furthermore, these data identify specific cellular responses to RHI that may direct future identification of diagnostic and therapeutic strategies for CTE.

## Abstract (web summary)

Repetitive head impacts from contact sports are associated with brain inflammation, vascular damage and neuron loss that are independent of hyperphosphorylated tau pathology.

## Main

Each year, millions of individuals are exposed to RHIs through contact sports, military service and domestic violence. These RHIs are often non-symptomatic and non-concussive, and can occur thousands of times per year, over the course of decades in some cases. CTE, a progressive tauopathy caused by exposure to RHI^1,2^, is observed in individuals as young as 17. Risk for CTE in exposed individuals is associated with the number of years of exposure to RHI and the cumulative force of the hits endured^3,4^. Although much of the current research is focused on severe CTE in older individuals, a recent case series of 152 brains from donors under the age of 30 identified 63 brains with CTE, highlighting that RHI-driven disease is a pressing concern in the young population^2^. Currently, CTE can only be diagnosed post-mortem through identification of p-tau aggregates in neurons around blood vessels at the depth of the cortical sulcus. Our previous research suggests that microglia-mediated neuroinflammation occurs prior to the deposition of p-tau^5^. Other work has demonstrated that RHI exposure is associated with astrocytic activation, white matter inflammation and damage, blood–brain barrier (BBB) breakdown, serum protein leakage and increases in vascular density in the CTE brain^5–9^. These cellular changes occur prior to overt neurodegeneration and are likely to drive many of the early clinical impairments that are not explained by the occurrence and extent of p-tau pathology. However, studies examining the full extent of these cellular phenotypes have been limited. A detailed characterization of the early cellular changes in young RHI-exposed athletes is necessary to understand the pathogenic mechanisms in CTE and to identify novel biomarkers or therapeutic targets relevant to early disease stages.

## Cell-type analysis across groups

**Fig. 1:**
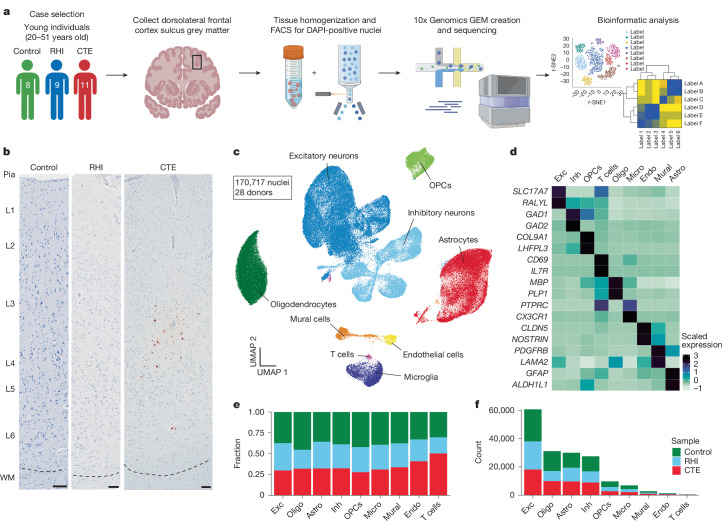
Cell-type identification and cell proportion analysis across pathological groups. **a**, Experimental workflow. Images created in BioRender. Cherry, J. (2025) https://BioRender.com/5kj3gsd. The workflow was run once for each sample. FACS, fluorescence-activated cell sorting; GEM, gel bead in emulsion. **b**, AT8 immunohistochemistry of dorsolateral frontal cortex depth of sulci. The dashed line represents the grey matter–white matter (WM) interface. Scale bars, 100 μm. **c**, Uniform manifold approximation and projection (UMAP) analysis of nuclei from all donors labelled for cell type based on cell-type marker expression. OPCs, oligocendrocyte precursor cells. **d**, Expression of cell-type markers across cell-type clusters in **c**. Astro, astrocytes; Endo, endothelial cells; Exc, excitatory neurons; Inh, inhibitory neurons; Micro, microglia; Oligo, oligodendrocytes. **e**, Stacked bar plot of pathological group fractions within cell-type clusters. **f**, Stacked bar plot of cell-type counts coloured by pathological group.

**Extended Data Fig. 1:**
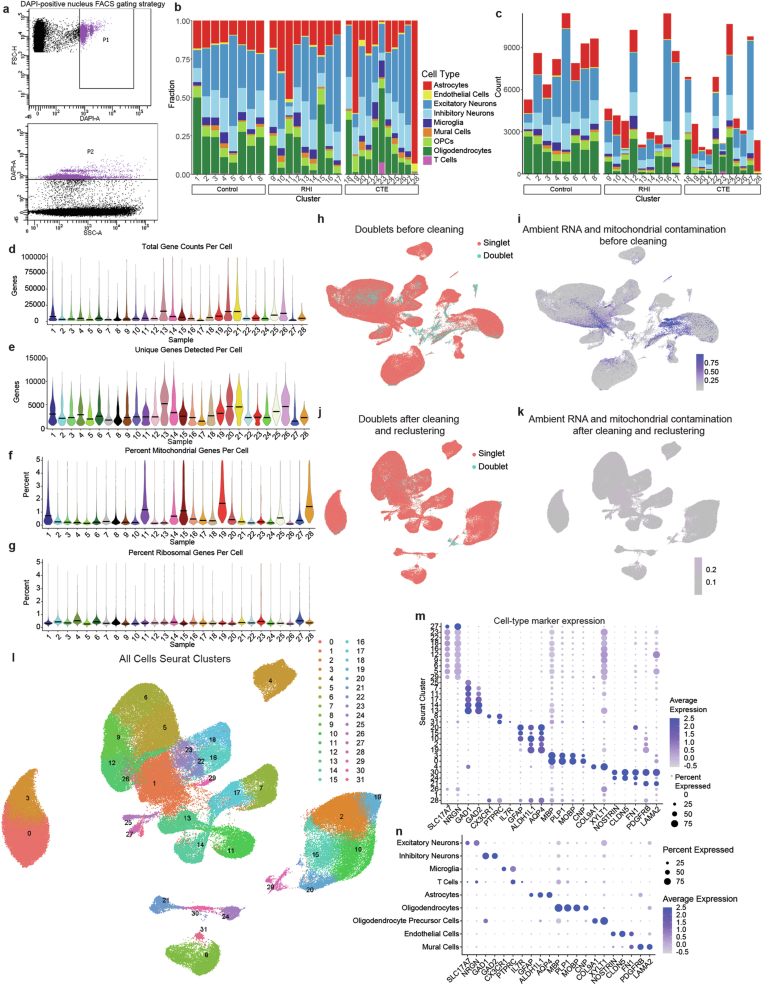
Dataset quality control and cell type marker validation. **a**. Fluorescence activated cell sorting gating strategy of DAPI-positive nuclei. **b**. Stacked bar plot representing the proportion of cell type per donor. **c**. Stacked bar plot representing the cell type counts per donor. **d-e**. Violin plots for each donor of (**d**) total gene counts per cell, (**e**) unique genes detected per cell, (**f**) percent of mitochondrial genes detected per cell, and (**g**) percent ribosomal genes detected per cell. Line represents median. **h, i** UMAP of full dataset before cleaning colored by (**h**) doublet or singlets or (**i**) mitochondrial contamination. **j, k**. UMAP of full dataset after cleaning colored by (**j**) doublets or singlets or (**k**) mitochondrial contamination. **l**. UMAP of full dataset colored by Seurat clusters. **m**. Dot plot of cell type marker expression across Seurat clusters depicted in (**l**). **n**. Dot plot of cell type marker expression in annotated cell type clusters.

**Extended Data Fig. 2:**
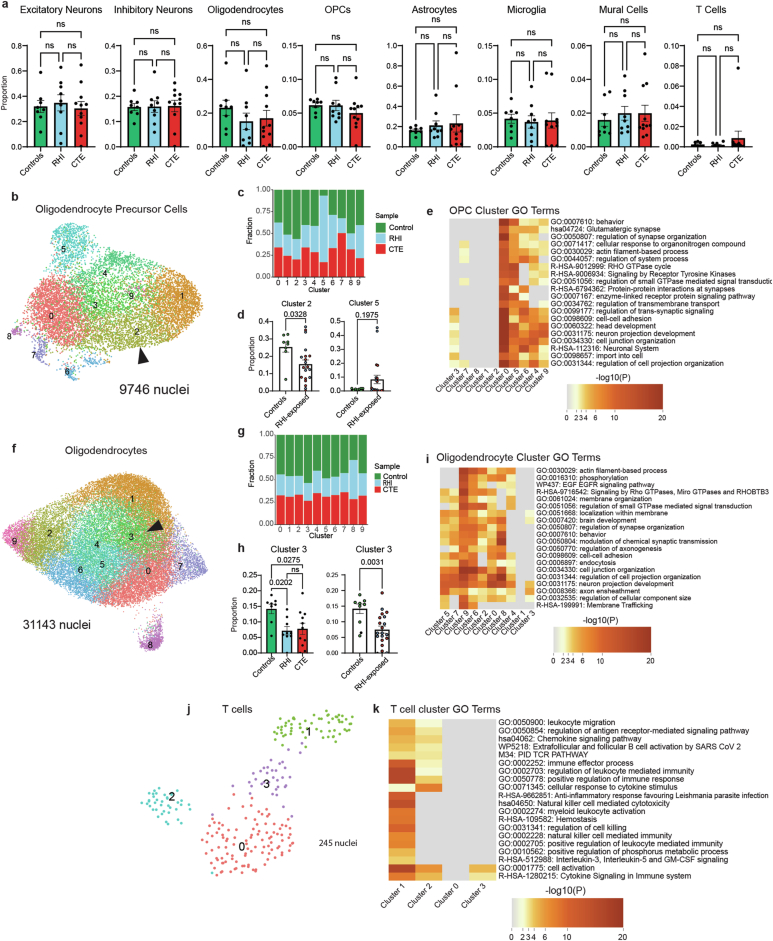
Cell type proportions, OPCs, Oligodendrocytes, and T-Cells. **a**. Bar plots of overall cell type proportions across pathological groups with each dot representing a sample, bars represent the mean, error bars represent standard error of the mean. Statistical analysis performed by ANOVA with Bonferroni correction. Test was two-tailed. n = 28 individuals. **b**. UMAP depicting OPCs colored by Seurat clustering, solid arrow indicating RHI/CTE depleted cluster. **c**. Stacked bar plot showing OPC Seurat cluster distribution across pathological groups. **d**. Bar plots showing OPC cluster distribution across control and pathological group or control and RHI-exposed samples, bar represents mean, error bars show standard error of the mean. Statistical analysis performed by ANOVA with Bonferroni correction (left) and two-tailed Mann-Whitney U test. n = 28 individuals. **e**. Heatmap showing GO analysis of OPC cluster DEGs. Statistics generated with GSEA and single-tailed hypergeometric test with Benjamini-Hochberg multiple hypothesis correction. **f**. UMAP showing oligodendrocytes colored by Seurat cluster, solid arrow indicates RHI and CTE depleted cluster. **g**. Stacked bar plot showing oligodendrocyte pathological group distribution per Seurat cluster. **h**. Bar plots representing cluster distribution across pathological groups or control and RHI-exposed samples. Bar represents mean, error bar represents standard error of the mean. Statistical analysis performed by ANOVA with Bonferroni correction (left) or two-tailed t-test (right). n = 28 individuals. **i**. Heatmap showing GO analysis of oligodendrocyte cluster DEGs. Statistics generated with GSEA and single-tailed hypergeometric test with Benjamini-Hochberg multiple hypothesis correction. **j**. UMAP showing T cells colored by Seurat cluster. **k**. Heatmap of GO analysis of T cell cluster DEGs. Statistics generated with GSEA and single-tailed hypergeometric test with Benjamini-Hochberg multiple hypothesis correction.

To identify the earliest RHI-driven changes, we performed single-nucleus RNA sequencing (snRNA-seq) using autopsy-confirmed frozen human brain tissue from 28 young individuals. Eight non-RHI-exposed controls, 9 RHI-exposed individuals without CTE pathology (RHI-only) and 11 RHI-exposed individuals with diagnosed CTE stage 1 or 2 (CTE) were included (Fig. 1a and Supplementary Tables 1 and 2). Of the individuals with RHI exposure, all but one individual played American football (hereafter referred to as football). The remaining individual played soccer (Supplementary Table 2). CTE diagnosis was performed by a neuropathologist and based on the presence of CTE pathognomonic p-tau lesions^10^ (Fig. 1b). Grey matter sulcus from the dorsolateral frontal cortex, one of the first brain regions affected in CTE, was processed for snRNA-seq (Fig. 1a). After quality control and filtering, 170,717 nuclei of sufficient quality were clustered into 31 initial clusters and labelled on the basis of their expression of known cell-type markers^11,12^ (Fig. 1c and Extended Data Fig. 1a–n). All major cell types were identified. Compositional analysis with scCODA demonstrated no significant differences in cell-type abundance across pathological groups^13^ (Fig. 1d–f and Extended Data Fig. 2a). Out of all major cell types, minimal RHI-associated changes were observed in oligodendrocytes and oligodendrocytes precursor cells (Extended Data Fig. 2b–i), probably resulting from the grey matter focus of the current study. We thus elected to focus further analyses on microglia, astrocytes, endothelial cells and neurons, consistent with previous studies^5,7,8,14^.

## RHI induces distinct microglia subtypes

**Fig. 2:**
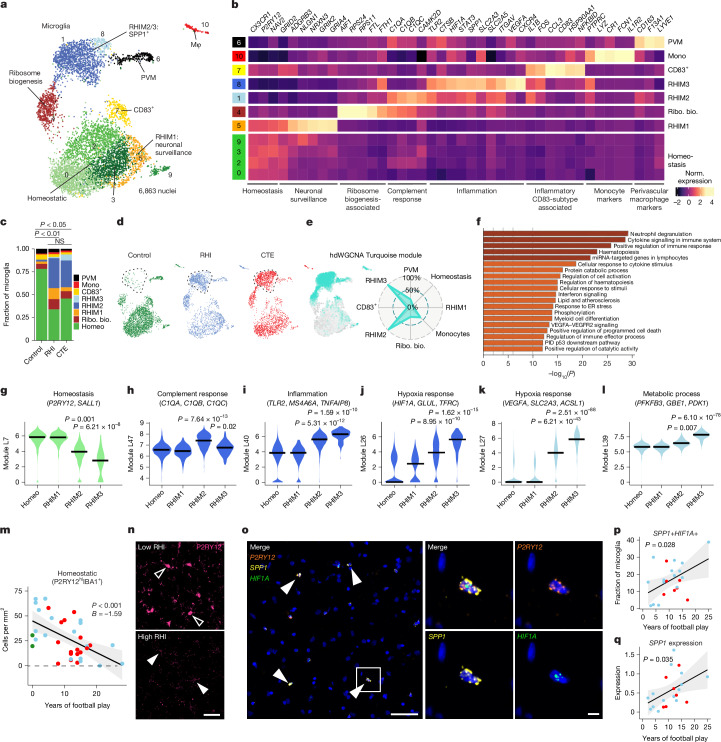
RHI exposure induces distinct microglial phenotypes. **a**, UMAP of microglia coloured by 11 Seurat clusters determined by unsupervised clustering. Mφ, macrophage; PVM, perivascular macrophage. **b**, Heat map of selected cluster DEGs annotated by function. Mono, monocyters; Ribo. bio., ribosome biogenesis; norm., normalized. **c**, Proportion of microglial subtypes per pathological group. Statistical analysis was performed using a chi-squared test. *n* = 28 individuals. Tests were two-tailed. Homeo, homeostasis. **d**, UMAP of each pathologic group. The dotted line depicts RHIM2/3 subtypes. **e**, hdWGCNA module analysis showing the Turquoise module localization to the RHIM2/3 subtype. **f**, GO analysis of the hdWGCNA Turquoise module. Statistics generated using gene set enrichment analyses (GSEA) and single-tailed hypergeometric test with Benjamini–Hochberg multiple hypothesis correction. ER, endoplasmic reticulum; miRNA, micro RNA; PID, Pathway Interaction Database. **g**–**l**, Violin plots of the Celda gene modules homeostasis (**g**), complement response (**h**), inflammation (**i**), hypoxia response (**j**), hypoxia response (**k**) and metabolic process (**l**). Colour represents the cellular subtype associated with the module. The black line represents the median. Statistical analysis performed by linear mixed effects modelling, correcting for patient-specific effects. Tests were two-tailed. *n* = 28 individuals. **m**, Quantification of grey matter sulcal homeostatic microglia (P2RY12^hi^IBA1^+^) with years of football play, coloured by pathological group. Statistical analysis performed by linear regression with age as a covariate. Each dot represents an individual donor. The black line represents linear model regression; the grey region shows the 95% confidence interval. The test was two-tailed. *n* = 37 individuals. **n**, Representative image of P2RY12 immunofluorescent labelling. Open arrowheads depict cells exhibiting high P2RY12 expression. Solid arrowheads depict cells exhibiting low P2RY12 expression. Scale bar, 50 μm. **o**, Representative image of *SPP1*+*HIF1A*+*P2RY12*+ microglia. Solid arrowheads indicate triple-positive cells. The white box indicates the inset displayed on the right. Scale bars: left, 50 μm; right, 5 μm. **p**,**q**, Quantification of the *SPP1*+*HIF1A*+ microglial fraction (**p**) and microglial *SPP1* expression (**q**) in the grey matter sulcus with years of football play. Each dot represents an individual donor. *n* = 22 individuals. Coloured by pathological group status. Statistical analysis performed by linear regression. The test was two-tailed. The black line represents linear model regression; the grey region shows the 95% confidence interval.

On the basis of previously demonstrated involvement of microglial inflammation in CTE and its important role in neurodegeneration, we examined changes in microglial gene expression^5^. Analysis of 6,863 microglial cells revealed 11 unique clusters (Fig. 2a). The microglia cluster size is consistent with other published studies and believed to be appropriately powered^11^. Cluster 10 contained 263 cells and expressed the perivascular macrophage genes *CD163*, *F13A1* and *LYVE1*, and cluster 6 was composed of 108 cells that expressed the peripheral monocyte genes *PTPRC*, *LYZ* and *CR1* as previously observed^11,15,16^ (Fig. 2b).

**Extended Data Fig. 3:**
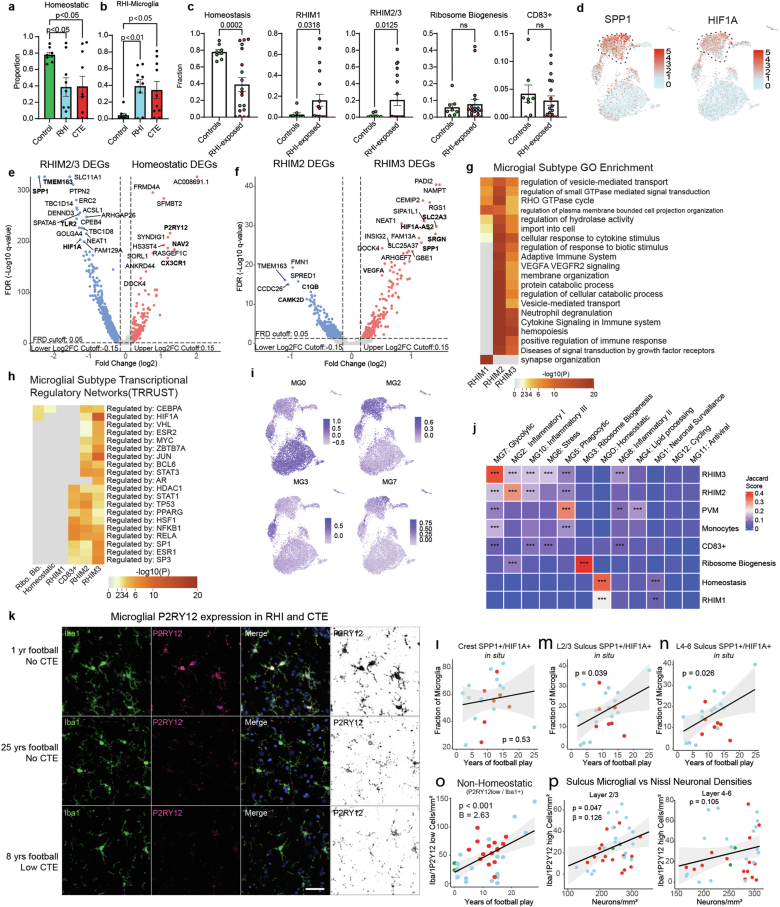
Microglial cluster GO analysis, histology, and validation. **a,b**. snRNAseq fraction of (a) homeostatic and (b) RHI microglia. Statistics performed by ANOVA with Bonferroni correction. Bar represents mean, error bars show SEM. Tests were two-tailed. n = 28 individuals. **c**. Microglial subtypes across control compared to RHI-exposed individuals. Statistical analysis performed by two-tailed t-test or Mann Whitney U test with Welch correction. Bar represents mean, error bars show SEM. n = 28 individuals. **d**. UMAPs of SPP1 and HIF1A microglial expression. Dashed lines indicate RHIM2/3. **e,f**. Volcano plots showing DEGs between RHIM2/3 and homeostatic microglia (e) and RHIM2 and RHIM3 (f). n = 28 individuals **g,h**. Heatmap of (g) GO analysis of RHI microglia and (h) transcriptional regulatory network analysis of microglial subtype DEGs. Statistics generated with GSEA and single-tailed hypergeometric test with Benjamini-Hochberg multiple hypothesis correction. **i**. UMAPs depicting microglia module scores of microglial subtypes from Sun et al. **j**. Heatmap depicting Jaccard score similarity analysis between Sun et al. and current study microglial DEGs. Significance denoted by **p < 0.01, ***p < 0.001. Statistical analysis performed using GeneOverlap package and Jaccard analysis settings. **k**. Representative images of P2RY12, Iba1. Scale bar, 50 μm**. l, m, n**. SPP1 + /HIF1A+ microglial fraction in the grey matter (l) crest, (m) L2/3 Sulcus (n) layers 4–6 sulcus colored by pathological group compared to years of football play. Statistical analysis performed by linear regression. Black line represents linear regression, grey shows 95% confidence interval. Test was two-tailed. n = 22 individuals. **o**. P2RY12 low/Iba1+ microglial densities in the grey matter sulcus compared to years of football play. Statistics with linear regression with age included as a covariate. Test was two-tailed. n = 37 individuals. **p**. Homeostatic microglial densities compared to Nissl+ neuronal densities in layers 2/3 (left) and layers 4–6 (right). Each dot represents a single individual. Statistical analysis performed by linear regression with age as a covariate. Tests were two-tailed. n = 37. Black line represents linear regression, grey shows 95% confidence interval.

Clusters 0, 2, 3 and 9 expressed the classical microglial homeostatic genes *CX3CR1*, *P2RY12* and *NAV2*, and were labelled as homeostatic microglia. Homeostatic clusters were significantly enriched for nuclei from control individuals compared with RHI-only or CTE individuals, but there was no significant difference between RHI-only and CTE individuals (control versus RHI-only: *P* = 0.048; control versus CTE: *P* = 0.047; RHI-only versus CTE: *P* > 0.99; Extended Data Fig. 3a). Homeostatic microglial proportion decreased with increasing years of football play (*P* = 0.004, *β* = −12.79). Cluster 7 highly expressed *CD83*, *CCL3* and *HSP90AA1*, reminiscent of a possible pro-resolving phenotype that was recently identified in Alzheimer’s disease^17^. Cluster 4 had the highest differential gene expression of *AIF1* (encoding IBA1) across clusters and was characterized by expression of the iron-associated genes *FTL* and *FTH* (also known as *FTH1*), and ribosome-associated genes such as *RPS24* and *RPS11* (Fig. 2b).

**Extended Data Fig. 4:**
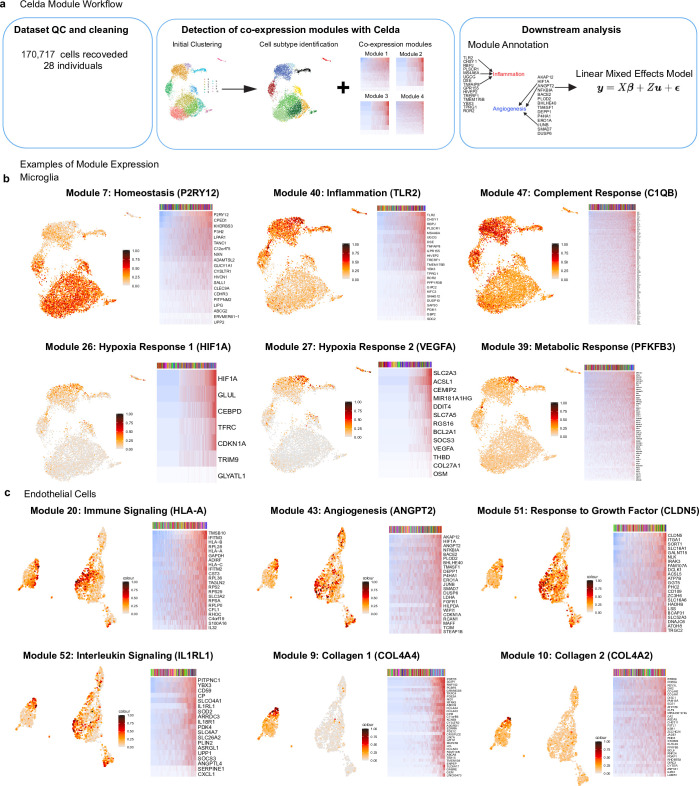
Celda module workflow and cell type expression. **a**. Celda module workflow diagram. **b**. Examples of Celda module expression in microglia. UMAPs show module expression, heatmaps show per-cell expression with genes listed on the right. **c**. Examples of Celda module expression in endothelial cells. UMAPs show module expression, heatmaps show per-cell expression with genes listed on the right. Genes can be viewed in Supplementary Tables 17–19.

The proportion of microglial subpopulations found in RHI-only and CTE individuals were significantly different from controls, with the emergence of clusters 1, 5 and 8 in RHI-only and CTE individuals (Fig. 2c and Extended Data Fig. 3a–c). For simplicity, these clusters were labelled RHI microglia (RHIM) 1 to 3. We performed gene module analysis with hdWGCNA and Celda to identify co-expression of possible cellular pathways across subclusters and linear mixed modelling statistical analysis to compare Celda gene module expression (Fig. 2d–l, Extended Data Fig. 4 and Supplementary Figs. 1–3). The full list of differentially expressed genes (DEGs) and gene modules can be found in Supplementary Tables 9 and 17. The expression of homeostasis-associated gene modules was significantly decreased in RHIM2 and RHIM3 (Fig. 2g).

Cluster 5, RHIM1, expressed neuronal-associated genes such as *GRID2*, *GRIK2* and *GRIA4*, with top identified Gene Ontology (GO) terms including ‘synapse organization’ (Fig. 2b and Extended Data Fig. 3g). Previous work has found that satellite microglia (microglia that closely contact neurons) increase in number following TBI and modulate neuronal firing activity^18^.

Cluster 1, RHIM2, was nearly evenly enriched for RHI and CTE samples (50% versus 46%, respectively), whereas cluster 8, RHIM3, were mostly enriched for CTE samples (83%). Transcriptionally, RHIM2 and RHIM3 were similar, displaying features of an inflammatory microglial phenotype with expression of *SPP1*, *HIF1A*, *TLR2*, *IL1B* and *CTSB* (Fig. 2b and Extended Data Fig. 3d,e). *SPP1* has been described as a general marker of inflammatory or activated microglia, and has a potential role in synaptic engulfment in Alzheimer’s disease models^19^. SPP1 has also been described as an opsin for extracellular debris^20,21^. hdWGCNA module analyses identified gene sets that were enriched in RHIM2 and RHIM3 that were strongly associated with immune signalling (Fig. 2e,f). Further GO analysis of RHIM2 and RHIM3 DEGs identified ‘cytokine signalling in the immune system’, ‘positive regulation of immune response’ and ‘vesicle mediated transport’ (Extended Data Fig. 3g). Celda gene module analysis demonstrated an increase in inflammation, hypoxia and metabolic response in RHIM2 and RHIM3 compared with homeostasis clusters (Fig. 2h–l), providing orthogonal validation of GO and DEG analyses.

Some key differences were noted between RHIM2 and RHIM3. RHIM2 expressed *C1QA*, *C1QB*, *C1QC* and *CAMK2D*, which encode the components and downstream effector of the C1q complement cascade that is known to drive aberrant synaptic engulfment in the neurodegenerative brain^22^ (Fig. 2b and Extended Data Fig. 3f). Gene module analysis further highlighted an increase in complement response in RHIM2 compared with homeostatic microglia (Fig. 2h). RHIM3 was characterized by upregulation of *HIF1A* and *VEGFA*, two central mediators of hypoxia, suggesting a potential response to or initiation of hypoxic conditions following RHI (Extended Data Fig. 3f). *HIF1A* also acts as a transcriptional regulator of many downstream inflammatory genes, and analysis of the transcriptional regulatory networks enriched in each cluster showed that RHIM3 expressed many genes regulated by HIF1A^23^ (Extended Data Fig. 3h).

To validate the reduction in the homeostatic microglial population, IBA1 and P2RY12 were co-immunolabelled and quantified in the sulcus of 35 individuals with 0 to 25 years of football play with or without CTE. Microglia were divided according to high or low P2RY12 expression. Homeostatic microglial densities (P2RY12^hi^IBA1^+^) were significantly decreased with increasing years of football play (*P* < 0.001; Fig. 2m,n). Concurrently, non-homeostatic microglia (P2YR12^low^IBA1^+^) cells were positively correlated with increasing years of football play (*P* < 0.001; Extended Data Fig. 3k,o). Mirroring the snRNA-seq results, CTE status was not significantly associated with homeostatic microglial densities when years of exposure were accounted for.

To verify the presence of RHIM2 and RHIM3 cells and their relationship to pathology, we performed in situ hybridization to label microglia that express the RHIM2 and RHIM3 marker genes *SPP1* and *HIF1A* (Fig. 2o). *P2RY12* was used as a marker for microglia as *AIF1* (encoding IBA1) exhibits low expression at the mRNA level, as evidenced in a previous study^23^ and the present snRNA-seq data. We quantified *SPP1*-expressing and *HIF1A*-expressing (*SPP1*+*HIF1A*+) microglia across 21 individuals with 2–25 years of football play with and without CTE (Fig. 2p,q). The number of *SPP1*+*HIF1A*+ microglia in the cortical sulcus increased significantly with increasing years of football play (*P* = 0.028; Fig. 2p). There was no association between *SPP1*+*HIF1A*+ microglia in the nearby cortical crest, suggesting a regional specificity of this inflammatory phenotype (*P* = 0.53; Extended Data Fig. 3l). We determined the layer specificity of *SPP1*+*HIF1A*+ microglia, separating superficial and deep layers of the cortical sulcus. The number of *SPP1*+*HIF1A*+ microglia increased in both superficial layers 2–3 (*P* = 0.039) and deeper layers 4–6 (*P* = 0.026) (Extended Data Fig. 3m,n). This suggests that although the microglial inflammation is specific to the sulcus, there was no layer-wise specificity of this phenotype. Additionally, microglia increased expression of *SPP1* with increasing years of football play (*P* = 0.035; Fig. 2q). CTE status (*P* = 0.34) and tau burden (*P* = 0.12) did not associate with the prevalence of *SPP1*+*HIF1A*+ microglia.

**Extended Data Fig. 5:**
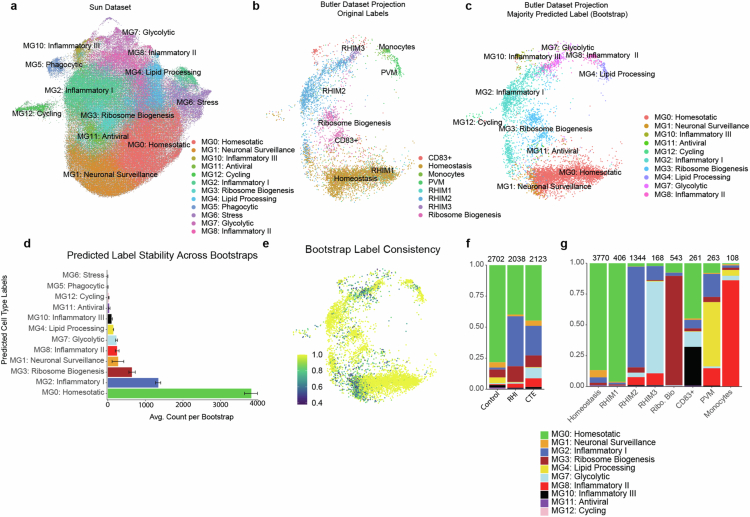
Microglia comparison with Sun et al. dataset. **a**. UMAP depicting microglia from Sun et al. dataset with original labels. **b**. Microglia from Butler et al. dataset projected onto Sun dataset UMAP space with original labels. n = 28 individuals. **c**. Microglia from Butler et al. dataset projected onto Sun dataset UMAP space with predicted Sun labels. n = 28 individuals. **d**. Bar plot depicting result of boostrapping, with number of predicted microglial labels and error bars depicting bootstrap confidence. **e**. UMAP depicting Butler microglia projected onto Sun dataset UMAP space colored by label consistency throughout bootstrapping. **f**. Stacked barplot depicting proportion of Butler microglia with Sun dataset labels across pathological groups. **g**. Stacked barplot depicting proportion of predicted labels across original labels within Butler dataset microglia showing fidelity across original labels and predicted labels.

Next, we sought to compare our microglial populations to those described in published datasets; notably, Sun et al.^23^ published a dataset with more than 100,000 microglia from more than 400 individuals. First, we performed Jaccard similarity scoring and confirmed significant correlation of the RHIM2 and RHIM3 clusters with inflammatory, stress, phagocytic and glycolysis-associated gene sets^23^ (Extended Data Fig. 3i,j). Next, we projected our data onto the dataset and performed bootstrapping to predict labels and confidence scores to better analyse the consistency of subtypes (Extended Data Fig. 5). Overall, we observed significant similarity between select subpopulations, further validating our subtypes.

Overall, these results suggest that RHI exposure induces an increase in neuronal surveillance and inflammatory microglial transcriptomic states before the onset of CTE. Inflammatory microglia are localized specifically at the sulcus in RHI-exposed individuals. These microglia may be involved in the initiation and maintenance of neuronal dysfunction, inflammation and angiogenic processes that are present in CTE.

## Astrocytic responses to RHI

**Extended Data Fig. 6:**
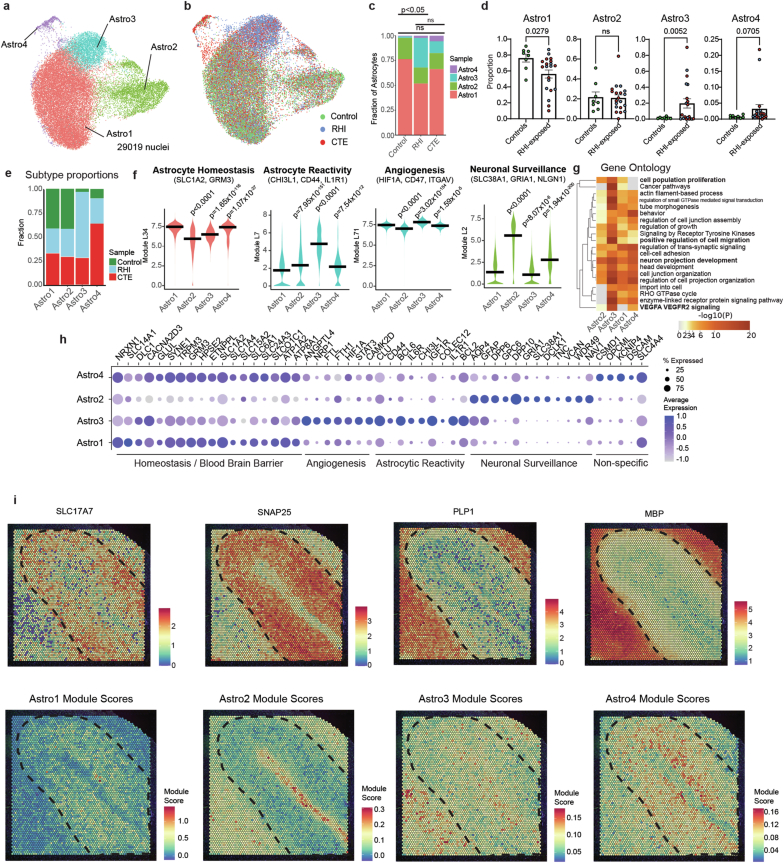
Astrocytic responses to head trauma. **a**. UMAP representing 4 astrocytic subtypes. **b**. UMAP from (a) colored by pathological group. **c**. Stacked bar plots showing astrocyte subtype distribution across pathological groups, statistics performed by chi-squared test. Tests were two tailed. n = 28 individuals. **d**. Bar plots showing astrocyte subcluster distribution in control and RHI-exposed samples, dots represent individual donors colored by pathological group identity. Bars represent mean, error bars represent standard error of the mean. Statistical analysis was performed using two-tailed Mann Whitney U-test. n = 28 individuals. **e**. Stacked bar plots showing pathological distribution across astrocyte subtypes. **f**. Violin plots showing Celda module expression across astrocyte subtypes. Black bar showing median statistic. Colored by astrocyte subtype most associated with specific module expression. Statistical analysis performed by linear mixed effects model. Tests were two-tailed. n = 28 individuals. **g**. Gene ontology analysis of astrocytic subtypes performed by Metascape. Statistics generated with GSEA and single-tailed hypergeometric test with Benjamini-Hochberg multiple hypothesis correction. n = 28 individuals. **h**. Dot plot representing expression of selected DEGs across astrocytic subtype and annotated by function. **i**. Projection of current astrocytic modules on to Visium spatial transcriptomic data. Top row of heatmaps show expression of white matter (PLP1, MBP) and grey matter (SLC27A7, SNAP25) genes. Dotted line indicates separation of grey and white matter. Heatmaps on the bottom row show expression of astrocyte subtype modules based on significantly upregulated genes in each subtype.

Astrocytes have a key role in brain homeostasis in tasks such as neuronal and BBB maintenance and become reactive following RHI exposure and in neurodegenerative disease^14,24^. We identified four subtypes of astrocytes, Astro1–Astro4, based on stratification of pathological group identity, DEG analysis and gene module analysis (Extended Data Fig. 6, Supplementary Figs. 5–7 and Supplementary Tables 10 and 19). Each subtype was also confirmed to be expressed in the cortical grey matter (Extended Data Fig. 6i). Although past work has suggested the importance of astrocytes in CTE, we observed a limited astrocytic response, with only one subtype being enriched for individuals with RHI (Astro3). Genes and gene modules that were upregulated in Astro3 were associated with astrocyte reactivity (*CHI3L1*, *CD44*, *CLU* and *BCL6*), inflammation (*IL6R* and *IL1R1*) and angiogenesis (*HIF1A*, *NRP1* and *ANGPTL4*) (Extended Data Fig. 6h). These findings suggest that although there is pronounced astrogliosis associated with end-stage CTE pathology, astrocytes might have a more subtle role in early disease.

## Endothelial angiogenic response to RHI

**Extended Data Fig. 7:**
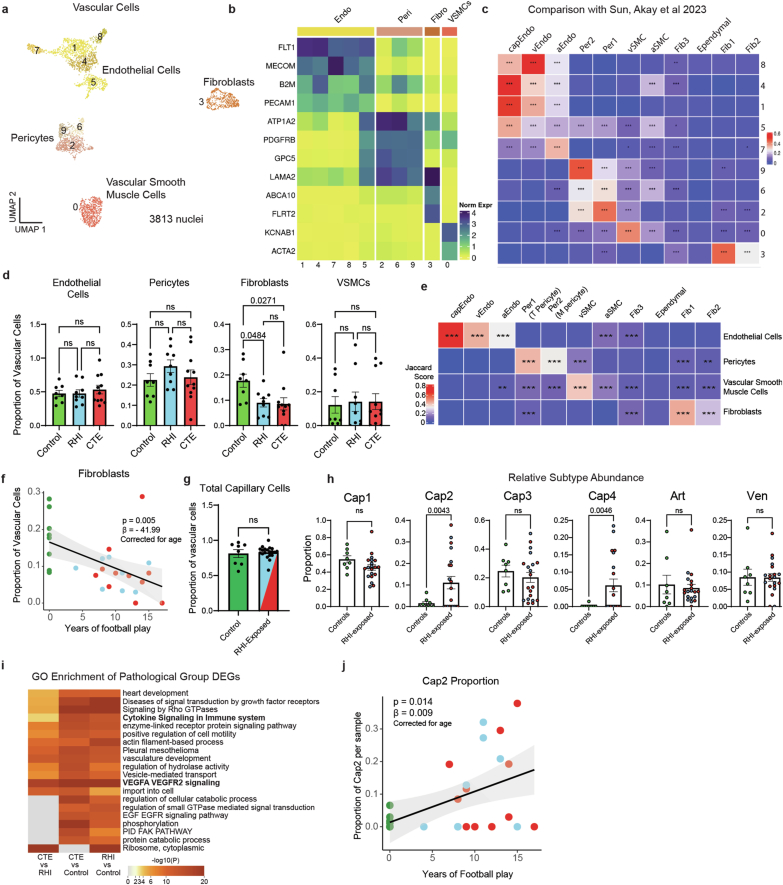
Vascular cell subtype identification and proportion analysis. **a**. UMAP showing all vascular cells colored by Seurat clustering. n = 28 individuals. **b**. Heatmap depicting vascular cell marker expression. **c**. Heatmap depicting Jaccard scoring of vascular cell Seurat cluster DEGs compared to Sun and Akay et al. vascular subtype DEGs. Significance denoted by **p < 0.01, ***p < 0.001. **d**. Bar plots depicting pathological group proportions of vascular subtypes, bar represents mean, error bar represents SEM, dots represent individual samples. Statistical analysis performed by ANOVA with Bonferroni correction. Tests were two-tailed. n = 28 individuals. **e**. Heatmap depicting Jaccard scoring of vascular cell subtype DEGs compared to Sun and Akay et al. vascular subtype DEGs. Significance denoted by ** p < 0.01, *** p < 0.001. **f**. Scatter plot of fibroblast proportion or Cap2 proportion compared to years of football play from snRNAseq dataset, colored by pathological group status. n = 28 individuals. Statistical analysis performed by linear regression with age as a covariate. Black line represents linear model regression, grey shows 95% confidence interval. Tests were two-tailed. **g, h**. Bar plots of total capillary and relative endothelial cell subtype distribution across control and RHI-exposed samples, dots represent individual donors and are colored by pathological group identity. n = 28 individuals. Bar indicates mean, error bars indicate standard error of the mean. Statistical analysis was performed by two-tailed Mann-Whitney U test. **i**. GO enrichment analysis of DEGs from depicted comparisons. Statistics generated with GSEA and single-tailed hypergeometric test with Benjamini-Hochberg multiple hypothesis correction. n = 28 individuals. **j**. Proportion of cap2 cells in each individual compared to years of football. Statistics generated with linear regression correcting for age. Black line represents linear model regression, grey shows 95% confidence interval. Each dot represents a single individual. Tests were two-tailed. n = 28 individuals.

Next, owing to the key involvement of vascular dysfunction in CTE, we characterized the vascular response to RHI exposure^8,9^ (Extended Data Fig. 7a). We used known cell-type markers and comparison with published dataset markers to identify 1,762 endothelial cells, 913 pericytes, 487 fibroblasts and 651 vascular smooth muscle cells^25^ (Extended Data Fig. 7b,c,e). Of these cell types, only fibroblasts displayed significant changes in total proportion across pathological groups, decreasing from controls to RHI (*P* = 0.048) and CTE (*P* = 0.027), with loss associated with years of football play (Extended Data Fig. 7d,f). Endothelial cells were further labelled for arterial, venous and capillary cells by comparing expressed genes with published datasets^25^ (Extended Data Fig. 7c,e). Capillary cells were then labelled Cap1–Cap4. Cap3 and Cap4 (Seurat Cluster 5; Extended Data Fig. 7b,c) exhibited a slightly different transcriptomic profile with higher expression of collagen-associated genes and showed overlap in expression of pericyte genes, thereby representing a potential transitional cell state but with greatest fidelity to endothelial cell expression (Extended Data Fig. 7c,e).

**Fig. 3:**
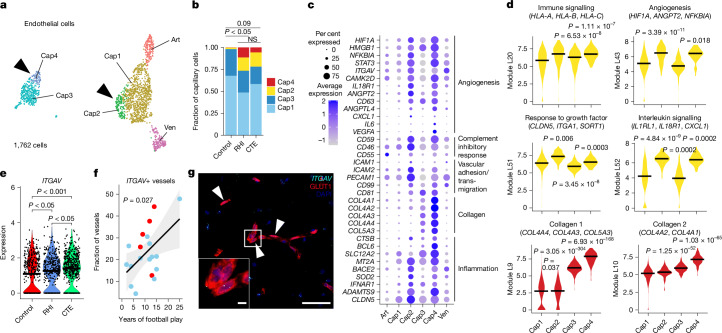
Endothelial angiogenic responses to RHI. **a**, UMAP analysis of endothelial cells coloured by endothelial cell subcluster. Solid arrowheads indicate clusters that are enriched in RHI and CTE. Art, arterial; Ven, venous. **b**, Stacked bar plots of capillary subtype abundance across pathological groups. Statistical analysis performed using a chi-squared test. Tests were two-tailed. **c**, Dot plot of selected DEGs that are upregulated in RHI and CTE across endothelial subtypes, annotated for function. **d**, Violin plots of Celda module expression across capillary subtypes. Black bars indicate the median statistic from ggsignif. Statistical analysis performed with linear mixed effects model, accounting for sample variability and comparing Cap2 and Cap3 with Cap1. Tests were two-tailed. *n* = 28 individuals. **e**, Violin plot of *ITGAV* expression across pathological groups. Each dot represents a cell. Statistical analysis performed by Wilcoxon test from ggsignif. Test was two-tailed. *n* = 28 individuals. **f**, Scatter plot of the *ITGAV*+ vessel fraction in the grey matter sulcus with years of football play, coloured by pathological group status using in situ hybridization. Each dot represents an individual donor. Statistical analysis performed by linear regression. *n* = 19. The line represents linear model regression; the grey region shows the 95% confidence interval. **g**, Representative image of an *ITGAV*+ vessel. Solid arrowheads indicate the *ITGAV*+ vessel. The white box indicates the region in the inset. Scale bars: left, 5 μm; right, 50 μm. Each individual sample was stained and analysed once.

The proportion of endothelial cell subtypes differed significantly between RHI and control individuals and trended towards a difference between control and CTE individuals (Fig. 3a,b). No difference was observed between RHI and CTE individuals (Fig. 3b). Two populations of capillary cells, Cap2 (*P* = 0.0043) and Cap4 (*P* = 0.0046) were enriched for RHI and CTE samples (Extended Data Fig. 7h). The Cap2 cell fraction also increased with increasing years of football play (*P* = 0.014; Extended Data Fig. 7j). No differences were observed in total capillary cells in RHI and CTE compared with controls (Extended Data Fig. 7g). Several canonical angiogenesis-associated genes such as *HIF1A*, *ANGPT2*, *ANGPTL4*, *STAT3*, *CAMK2D* and *NFKBID* were significantly upregulated in Cap2 and Cap4, suggesting that capillary cells in RHI-exposed groups may be responding to a local hypoxic environment (Fig. 3c,d). Three major complement regulatory genes—*CD59*, *CD55* and *CD46*—which inhibit complement-mediated cell lysis, were upregulated indicating a potential response to locally increased levels of complement (Fig. 3c). Expression of the vascular adhesion and transmigration-associated genes *ICAM1*, *ICAM2*, *PECAM1* and *CD99* was increased in Cap2 and Cap4, indicating an increased potential for entry of monocytes, T cells, neutrophils or other peripheral cell types across the endothelium (Fig. 3c). Cap4 also exhibited high expression of collagen genes (Fig. 3c). We performed module co-expression analysis using Celda and hdWGCNA to identify co-expressed genes and possible cellular pathways across endothelial subsets (Supplementary Figs. 8–10). Statistical linear mixed modelling demonstrated that modules related to immune signalling, angiogenesis, response to growth factors and collagen were significantly upregulated in Cap2 and Cap4 subsets (Fig. 3d, Supplementary Fig. 8 and Supplementary Tables 11 and 18). hdWGCNA also identified enrichment of collagen-associated genes in Cap4 as additional validation (Supplementary Table 18c and Supplementary Fig. 9, blue module). GO analysis identified VEGFA signalling, cytokine signalling and vasculature development as significantly upregulated terms in RHI-exposed endothelial cells (Extended Data Fig. 7i). We identified *ITGAV* as an endothelial gene that was significantly increased in Cap2 cells and in RHI compared with control and CTE samples (Fig. 3e). To confirm its expression in the tissue, we performed in situ hybridization paired with GLUT1 immunohistochemistry to label vessels, and found an increase in the fraction of vessels expressing *ITGAV* with increasing years of football play (*P* = 0.027; Fig. 3f,g). Together, these data show that capillary cells undergo significant upregulation of angiogenesis and inflammation-associated genes along with an increase in basement membrane components, identifying pathways that may underlie the known microvascular dysfunction after RHI and in CTE^8,9^.

## Neuronal dysfunction and loss after RHI

**Fig. 4:**
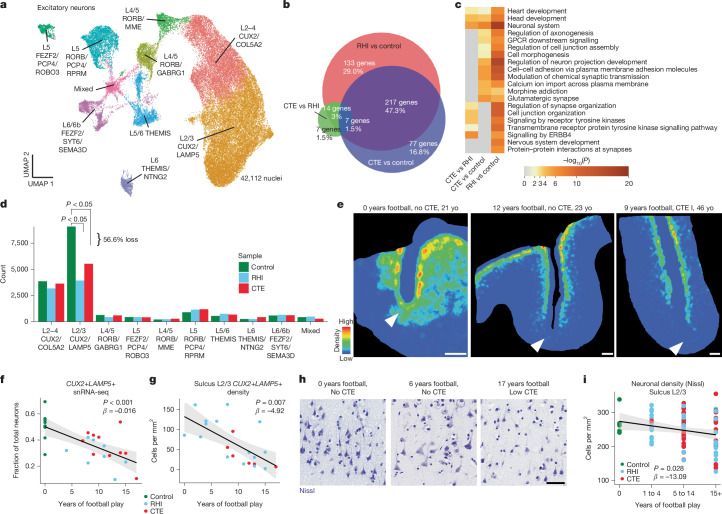
Synaptic transcriptomic changes and loss of sulcal excitatory layer 2/3 neurons. **a**, UMAP analysis of excitatory neurons coloured and labelled by layer subtype. **b**, Venn diagram depicting the overlap between DEGs from RHI versus control, CTE versus control and RHI versus CTE comparisons. *n* = 28 individuals. **c**, Heat map of GO terms identified in comparisons listed in **b**. Statistics generated using GSEA and single-tailed hypergeometric test with Benjamini–Hochberg multiple hypothesis correction. GPCR, G-protein-coupled receptor. **d**, Bar plot representing cell counts per pathological group for each excitatory neuron layer subtype. Statistical analysis performed by ordinary one-way analysis of variance (ANOVA) with Bonferroni correction. The test was two-tailed. *n* = 28 individuals. **e**, Representative density heat map of *CUX2*+*LAMP5*+ cells from in situ hybridization. Solid arrowheads indicate depth of the cortical sulcus. Red indicates high cellular density; blue indicates low cellular density. Scale bars, 1 mm. **f**, Scatter plot showing the fraction of *CUX2*+*LAMP5*+ neurons among total excitatory neurons in snRNA-seq data with total years of football play, coloured by pathological group identity. Dots depict individual donors, the black line represents general linear model regression; the grey region shows the 95% confidence interval. The test was two-tailed. *n* = 28 individuals. **g**, Scatter plot showing cell density of *CUX2*+*LAMP5*+ neurons in sulcal layer 2/3 from in situ hybridization coloured by pathological group identity, with years of football play. Dots depict individual donors; the black line represents general linear model regression; the grey region shows the 95% confidence interval. *n* = 28 individuals. Statistics performed by general linear regression. The test was two-tailed. **h**, Representative images of Nissl-stained neurons in superficial cortical layer 2/3. Scale bar, 50 μm. **i**, Scatter plot showing Nissl-stained neuronal densities across football exposure groups. Dots depict individual donors; the line represents general linear model regression; the grey region shows the 95% confidence interval. The test was two-tailed. *n* = 28 individuals.

**Extended Data Fig. 8:**
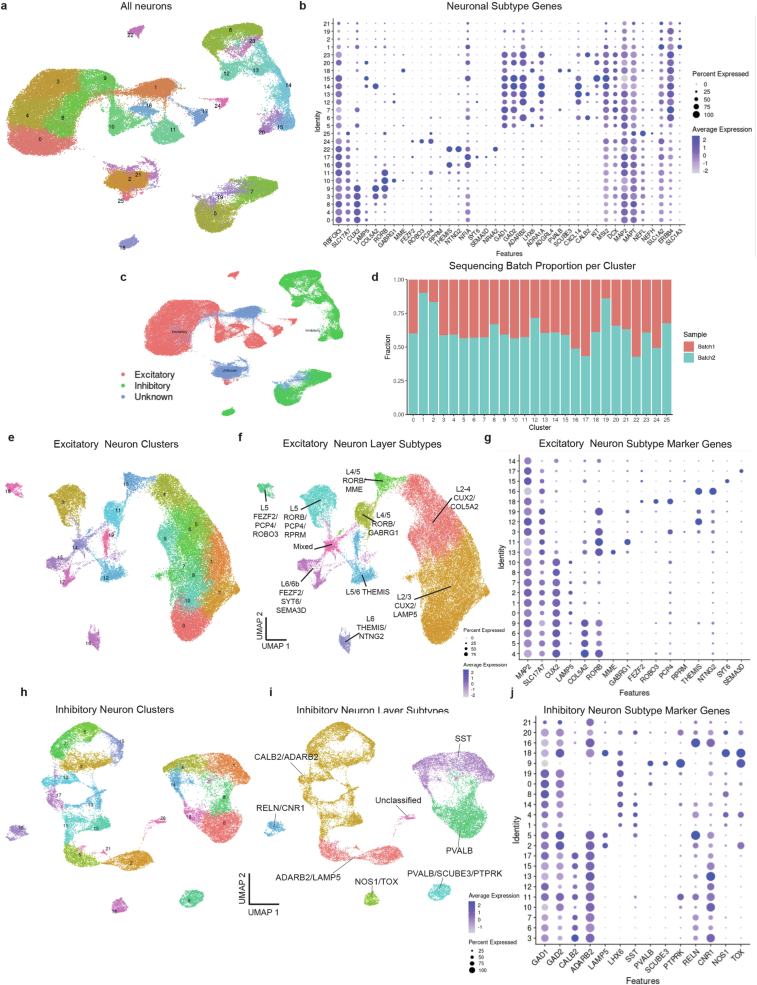
Neuronal layer subtype identification. **a**. UMAP depicting all neurons clustered together colored by Seurat cluster. n = 28 individuals. **b**. Dot plot of gene expression of inhibitory and excitatory neuron and astrocyte marker genes Seurat clusters from (a). **c**. UMAP from (a) colored by cell type determination. **d**. Stacked bar plot of sequencing batch distribution of Seurat clusters from (a). **e**. UMAP showing excitatory neurons colored by Seurat cluster. **f**. UMAP showing excitatory neurons colored by later subtype. **g**. Dot plot showing expression of excitatory neuron layer subtype genes in excitatory neuron Seurat clusters from (e). **h**. UMAP showing inhibitory neurons colored by Seurat cluster. **i**. UMAP showing inhibitory neurons colored by layer subtype. **j**. Dot plot showing expression of inhibitory neuron layer subtype genes across inhibitory neuron Seurat clusters from (h).

**Extended Data Fig. 9:**
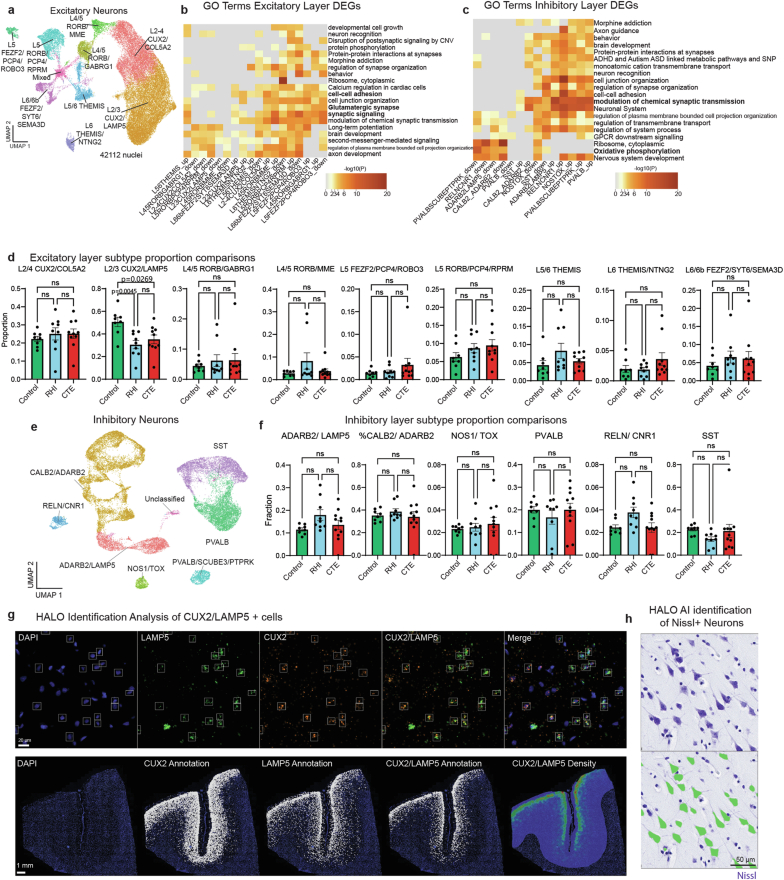
Neuron layer GO analysis, pathological group enrichment and RNAScope validation. **a**. UMAP depicting excitatory neurons colored by layer subtype. **b,c**. Heatmap showing GO analysis of (b) excitatory layer and (c) inhibitory layer up and downregulated DEGs. n = 28 individuals. Statistics generated with GSEA and single-tailed hypergeometric test with Benjamini-Hochberg multiple hypothesis correction. **d**. Bar plots of excitatory neuron layer proportions by pathological group. Bar represents mean, dots represent individual samples, error bars show standard error of the mean. Statistical analysis performed by ANOVA with Bonferroni correction. Tests were two tailed. n = 28 individuals. **e**. UMAP showing inhibitory neurons colored by layer subtype. **f**. Bar plots of inhibitory neuron layer proportions by pathological group. Bar represents mean, dots represent individual samples, error bars show standard error of the mean. Statistical analysis performed by ANOVA with Bonferroni correction. Tests were two tailed. n = 28 individuals. **g**. Representative image showing RNAScope in situ hybridization of CUX2/LAMP5 image analysis with correct anatomical layer-wise distribution. White squares showing HALO identification of double-positive cells. Scale bar = 1 mm. **h**. Representative image of Nissl+ staining and neuronal masking using HALO AI. Top box is the raw image and bottom box is AI generated mask over neurons. Scale bar = 50 µm.

Next, owing to the known dysfunction and degeneration of neurons and synaptic dysfunction following head trauma and in neurodegenerative disease, we examined neurons, labelling subclusters using known layer-specific markers^12,26–31^ (Fig. 4a and Extended Data Fig. 8). Forty-seven per cent of excitatory neuron DEGs were shared across RHI and CTE when compared with control, and only 6% were different between RHI and CTE, suggesting that the greatest changes in excitatory neuronal transcriptional profiles occur with initial exposure to RHI (Fig. 4b). Comparison of gene expression in RHI and CTE with controls and GO analysis of total neuronal population and layer-specific DEGs showed enrichment of ‘modulation of chemical synapses’ and ‘cell–cell adhesion’ processes in both analyses (Fig. 4c and Extended Data Fig. 9b). Genes associated with synaptic transmission such as *SYN3*, *SNAP91*, *NRG1*, *HSP12A1* (a member of the Hsp70 gene family), and genes encoding matrix binding proteins such as *CNTN5* and *CLSTN2* were upregulated across several excitatory neuron layers. There were 40% fewer layer-specific DEGs in inhibitory neurons than in excitatory neurons, with only 184 DEGs specific to RHI-exposed groups compared with controls. GO analysis of inhibitory neuron layer-specific DEGs showed common upregulation of synapse-associated genes such as *SYN3* and *SYN2* across layers and downregulation of GABA receptor gene *GABRA1* (Extended Data Fig. 9c).

**Extended Data Fig. 10:**
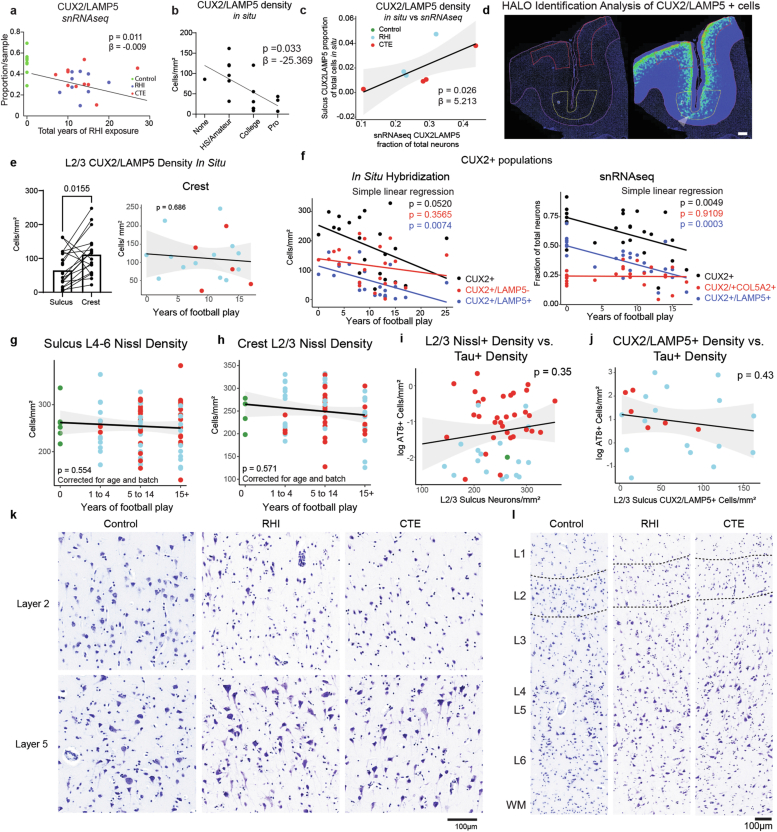
Layer 2/3 neurons are selectively lost in the grey matter sulcus and do not associate with tau pathology. **a**. CUX2/LAMP5 proportion from snRNAseq against total years of football play colored by pathological group. Statistical analysis performed by linear regression, depicted as black line. Test was two-tailed. n = 28 individuals. **b**. CUX2/LAMP5 density from in situ hybridization compared to highest level of football played. Statistical analysis performed by linear regression. Dots represent single individuals; line shows linear regression. Test was two-tailed. n = 23 individuals. **c**. CUX2/LAMP5 cells identified by in situ experiment compared to proportion of CUX2/LAMP5 neurons from snRNAseq experiment. Statistical analysis performed by linear regression, depicted as black line with 95% confidence intervals in grey. Test was two-tailed. n = 7 individuals. **d**. Representative image showing the annotation strategy used to identify the sulcus (yellow line) and crest (red line) layer 2/3. Scale bar = 1 mm. **e**. (left) Layer 2/3 CUX2+/LAMP5+ neuronal density in the sulcus and crest. Statistical analysis performed by paired t-test. Tests were two tailed. n = 18 individuals. (right) Layer 2/3 CUX2+/LAMP5+ neuronal density in the crest compared to years of football play, colored by pathological group. Statistical analysis performed by linear regression. Black line represents general linear model regression, grey shows 95% confidence interval. Tests were two tailed. n = 18 individuals. **f**. Total CUX2 populations and subpopulations in snRNAseq (right panel) and in situ hybridization (left panel) experiments compared to years of football play. Statistical analysis performed by linear regression and represented by lines. Tests were two-tailed. left panel n = 22 individuals, right panel n = 28 individuals. **g, h, i**. Scatter plots depicting (g) Sulcus layers 4–6 Nissl+ density compared to binned years of football play, (h) Crest layer 2/3 Nissl+ density compared to binned years of football play, (i) L2/3 Nissl+ density compared to log tau+ density, Statistical analysis performed by linear regression, (g, h) corrected for age and staining batch. n = 86 individuals. **j**. CUX2+/LAMP5+ density in situ compared to log AT8+ tau+ density, Colored by pathological group. Statistical analysis performed by linear regression. Test were two-tailed. Black line represents general linear model regression, grey shows 95% confidence interval. n = 22 individuals. **k, l**. Representative images of Nissl staining across cortical layers depicting neuronal loss in superficial layers in RHI and CTE individuals. Scale bars, 100 μm.

Since neurodegenerative processes and exposure to head trauma can be associated with neuronal loss, we investigated layer-specific cell composition in individuals with RHI and CTE compared with controls. No pathological group enrichment was found in inhibitory neurons (Extended Data Fig. 9e,f). However, differential abundance analysis of excitatory layer 2/3 *CUX2*+*LAMP5*+ neurons demonstrated a significant decrease in individuals with a history of RHI, regardless of CTE status (Fig. 4d and Extended Data Fig. 9d). These results were confirmed via multinomial Dirichlet multinomial regression to account for the compositional nature of snRNA-seq data^13^. RHI exposure individuals had an average of 56% fewer *CUX2*+*LAMP5*+ neurons than age-matched unexposed controls. When measured by proportion of total neurons, loss of *CUX2*+*LAMP5*+ neurons were also observed when comparing RHI (*P* = 0.0045) and CTE (*P* = 0.0269) individuals with controls (Extended Data Fig. 9d). Neuronal loss was associated with the number of years of playing football, or in the few cases with other types of contact sports play, total years of RHI exposure, independent of age at death (*P* < 0.001; Fig. 4f and Extended Data Fig. 10a).

To determine the spatial localization of the neuronal loss and validate the snRNA-seq results, we performed quantitative histology with RNAScope in situ hybridization using the excitatory layer 2/3 neuron markers *CUX2* and *LAMP5*. *CUX2*+*LAMP5*+ neuronal density at the sulcus was negatively associated with years of football play (*P* = 0.007, *β* = −4.92) and highest level of football played (*P* = 0.033, *β* = −25.34) (Fig. 4e,g and Extended Data Fig. 10b). *CUX2*+*LAMP5*+ cell density was significantly lower at the depth of the cortical sulcus compared with the nearby gyral crest, consistent with RHI-specific damage and CTE pathology^32^ (Fig. 4e and Extended Data Fig. 10d,e). *CUX2*+*LAMP5*+ cell densities at the crest were not associated with years of play, demonstrating a regional specificity of neuronal cell loss to the sulcus (*P* = 0.686; Extended Data Fig. 10e). *CUX2*+*LAMP5*− (putatively CUX2^+^COL5A2^+^) neurons were found intermixed with *CUX2*+*LAMP5*+ neurons throughout layers 2–4 and are putatively exposed to similar levels of mechanical forces due to adjacent anatomical location. However, neuronal loss was observed to be specific to *CUX2*+*LAMP5*+ excitatory neurons across in situ and snRNA-seq experiments, suggesting a specific susceptibility of this population to RHI exposure (Extended Data Fig. 10f).

To further validate the association between years of football play and neuronal loss, we determined total neuronal densities using Nissl staining of 86 young individuals with 0–28 years of football play. Individuals were grouped by 0, 1–4, 5–14 and 15 or more years of football play based on previously defined thresholds for CTE risk^3^. Layer 2/3 sulcal neuronal density significantly decreased with increased binned years of football play, independent of age at death (*P* = 0.028, *β* = −13.09; Fig. 4h,i). No association was found between years of football play and neuronal densities in deeper layers 4–6 (*P* = 0.554) or in layer 2/3 (0.571) in the crest (Extended Data Fig. 10g,h,k,l).

As p-tau deposition has been shown to associate with neuronal loss in neurodegenerative disease, we compared neuronal densities with p-tau pathology in adjacent sections. We found no association between neuronal loss and p-tau deposition, suggesting that neuronal loss occurs prior to and independent of pathologic protein deposition in early stages of disease (in situ hybridization: *P* = 0.387; Nissl staining: *P* = 0.825; Extended Data Fig. 10i,j).

Microglia contribute and respond to neuronal loss^33^. To investigate potential relationships between the observed neuronal loss and loss of microglial homeostasis, layer-wise homeostatic microglial populations (P2RY12^hi^IBA1^+^) from adjacent histological sections were compared to neuronal densities. Neuronal densities were significantly positively associated with homeostatic microglial populations in layers 2/3 (*P* = 0.047, *β* = 0.126; Extended Data Fig. 3p). By contrast, in layer 4–6 neuronal densities were not associated with homeostatic microglia populations (*P* = 0.105; Extended Data Fig. 3p), suggesting that loss of microglial homeostasis may be specifically localized to regions of neuronal loss.

Overall, these results provide evidence that exposure to RHI alone may drive significant neuronal loss and dysfunction, which may help explain early symptom onset in young athletes without the presence of significant p-tau pathology. Additionally, the relationship between neuronal loss and loss of microglial homeostasis point to potential mechanisms of or responses to neuronal loss.

## Ligand–receptor pair analysis

**Fig. 5:**
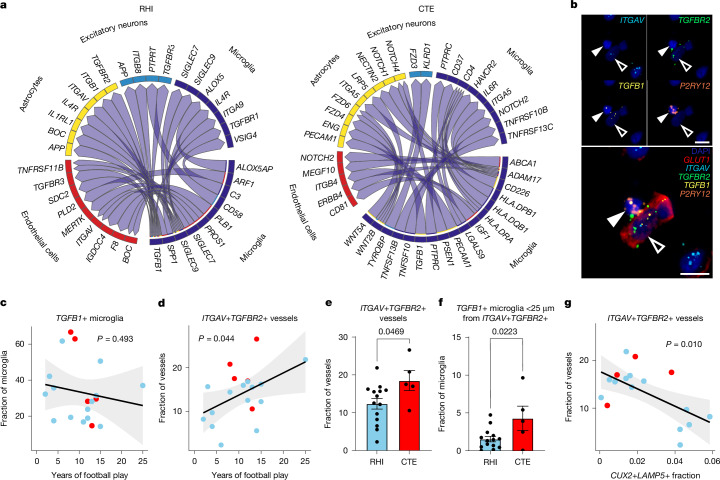
Ligand–receptor pair analysis in RHI exposure and CTE. **a**, Circos plots from MultiNicheNet analysis depicting microglia as sender cells. RHI comparison with control is labelled RHI; CTE comparison with RHI is labelled CTE. *n* = 28 individuals. **b**, RNAScope in situ hybridization depicting a *TGFB1*+ microglia (*P2RY12*; solid arrowheads) contacting a *ITGAV*+*TGFBR2*+ vessel (*GLUT1*; open arrowheads). Scale bars, 10 μm. **c**,**d**, Quantification of in situ hybridization of *TGFB1*+ microglia (**c**) and *ITGAV*+*TGFBR2*+ vessels (**d**) in the grey matter sulcus with years of football play, colour-coded by pathological group. Each dot represents an individual donor. Statistical analysis performed by simple linear regression. The black line represents general linear model regression; the grey region shows the 95% confidence interval. The test was two-tailed. *n* = 19 individuals. **e**, Bar plot representing *ITGAV*+*TGFBR2*+ vessels with CTE status. Statistical analysis performed using a two-tailed *t*-test. Data are mean ± s.e.m. The test was two-tailed. *n* = 19 individuals. **f**, The proportion of *TGFB1*+ microglia within 25 μm of a *ITGAV*+*TGFBR2*+ vessel with CTE status. Statistical analysis performed using a two-tailed *t*-test. *n* = 19 individuals. Data are mean ± s.e.m. **g**, Scatter plots depicting *ITGAV*+*TGFBR2*+ vessels in the grey matter sulcus with the fraction of *CUX2*+*LAMP5*+ neurons colour-coded by pathological group. Each dot represents an individual donor. Statistical analysis performed by simple linear regression. *n* = 17 individuals. The black line represents general linear model regression; the grey region shows the 95% confidence interval. The test was two-tailed.

To identify signalling pathways that may be involved in the cellular response to RHI exposure and CTE pathology, we performed ligand–receptor pair analysis using MultiNicheNet^34^. Two comparisons were run: RHI was compared with control (Fig. 5a, labelled RHI) to examine signalling occurring in the context of head trauma; and CTE was compared with RHI (Fig. 5a, labelled CTE) to investigate what signalling might be involved in the deposition of p-tau. In RHI-exposed individuals, microglial TGFB1 was identified as an important ligand, which signals to endothelial cells, astrocytes, neurons and other microglia through the TGFB1 receptors ITGAV, TGFBR2, TGFBR3 and TGFBR1. SIGLEC9 and SPP1 were also identified as major signalling hubs in RHI compared with control, implicating the RHIM2/3 phenotype in RHI-associated signalling. In CTE compared with RHI, the top microglial signalling pathways identified also included TGFB1 signalling and WNT2B and HLA-DRA signalling to astrocyte, microglia, endothelial cells and excitatory neurons. TGFB1 signalling has previously been implicated in the activation of neuroinflammation and induction of neuronal cell death in mild TBI^35^. Additionally, TGFB1 is involved with the fibrogenic response to mechanical stretch stimulus through ITGAV activation on endothelial cells and angiogenic responses through TGFBR2 signalling^36,37^.

We performed in situ hybridization analysis to label *TGFB1* in microglia, as well as *TGFBR2* and *ITGAV*, which encode two of the receptors identified in endothelial cells ligand–receptor pair analysis (Fig. 5b). We hypothesized that *TGFB1*+ microglia would increase in proximity to *ITGAV*+*TGFBR2*+ vessels to facilitate signalling. The prevalence of *TGFB1*-expressing microglia did not increase with the level of exposure (*P* = 0.493) or with CTE status (*P* = 0.493), however, we found an increase in *ITGAV*+*TGFBR2*+ vessels with increasing years of football play and with CTE status (*P* = 0.047) (Fig. 5c–e). Additionally, there was an increase in *TGFB1*+ microglia within 25 μm of *ITGAV*+*TGFBR2*+ vessels in CTE individuals compared with RHI-exposed individuals without CTE (Fig. 5f). The increase in microglia–endothelial cell pairs is likely to be driven by an increase in endothelial ITGAV and TGFBR2 expression as opposed to an increase in TGFB1+ microglia, concurring with data from the microglia and endothelial sections. We then compared the prevalence of *ITGAV*+*TGFBR2*+ vessels to *CUX2*+*LAMP5*+ neuronal populations in adjacent sections to identify potential relationships between the identified signalling pathway and the observed neuronal loss. We found that with decreasing neuronal populations in the grey matter sulcus, there was an increase in *ITGAV*+*TGFBR2*+ vessels (*P* = 0.010) (Fig. 5g). Overall, these findings identify a possible signalling pathway in the microglia–endothelial cell cross talk that may be implicated in the early pathological cascade of CTE pathology.

## Discussion

Here we utilize a combination of snRNA-seq, multiplex in situ hybridization and immunohistological analyses to describe and validate a unique dataset of young individuals with exposure to RHI. We describe distinct microglial and endothelial subsets that emerge following RHI and persist with CTE, correlate with years of contact sport play and associate with neuronal loss. Additionally, we observed a sulcus-specific loss of cortical layer 2/3 neurons that correlated with exposure to RHI prior to p-tau deposition. Finally, we identify a possible TGFβ signalling cascade between microglia and endothelial that might drive early pathogenesis.

Microglial, astrocytic and endothelial cell transcriptomic subtypes have been described in several neurodegenerative diseases and in severe traumatic brain injury, however to our knowledge, this study is the first to demonstrate such changes in a cohort of young individuals with exposure to repeated non-concussive head impacts. Of note, hypoxia-associated changes are present across these three cell types, suggesting an important role for vascular dysfunction and bolstering previous evidence of vascular remodelling in CTE. Forces from head trauma disproportionately affect blood vessels, causing a lasting endothelial response that affects BBB integrity and oxygen delivery in affected regions^32^. Activated endothelium, local hypoxia and a breached BBB may trigger a feedback loop, activating astrocytes and microglia with each head impact. Repeated blows to the head in short succession are likely to reactivate an already inflamed system, disallowing sufficient time for full repair, and preventing a return to homeostasis. This is substantiated by the increased microglial activation that is observed decades after retirement from contact sports and is found to correlate with the number of years of RHI exposure in this study and others^5^. Through this repetitive reactivation, the inflammatory response becomes self-sustaining and chronic, the mechanisms of which remain unclear. Another potential mechanism identified in this study included increased collagen expression in endothelial cells, a potential indicator of an early endothelial fibrotic response. The TGFB1–ITGAV/TGFBR2 signalling between microglia and endothelial cells that we identified through ligand–receptor analysis may represent a potential signalling pathway for the observed endothelial activation.

We observed an approximately 56% decrease in superficial layer 2/3 excitatory neurons in RHI-exposed individuals at the depths of the cortical sulci, the region known to sustain the most mechanical force upon head trauma, and the region of initial p-tau accumulation in CTE^32^. To our knowledge, this study is the first to demonstrate such a substantial loss of a specific neuronal subtype in young individuals driven solely by RHI exposure. This is especially concerning considering that several of the observed individuals had no neuropathologic protein deposition, suggesting that neurodegeneration might begin sooner than CTE onset. Recent studies have demonstrated cortical thinning in frontal regions of high school football players, and cortical thinning and neuronal loss in post-mortem individuals with CTE^38,39^. Neuronal loss might explain symptoms of traumatic encephalopathy syndrome, the clinical criteria for antemortem CTE diagnosis, in young athletes^2,40,41^. Layer 2/3 neurons make cortico-cortical connections and, in the frontal cortex, are associated with depressive behaviours and moderation of stress^42^. Notably, layer 2/3 neurons have been shown to be vulnerable in other neurodegenerative and psychiatric disorders and are susceptible to p-tau accumulation in Alzheimer’s disease^12,27^. Therefore, one may speculate that superficial layer 2/3 excitatory neurons are highly susceptible to damage regardless of source and our data captured the loss across a range of RHI doses. Vulnerability has been hypothesized to be caused by the longer projections being more susceptible to trauma-related diffuse axonal injury or the overall higher metabolic demand of these cells, however the exact mechanisms driving this susceptibility have yet to be explained. Although RHI damage is driving the early neuronal loss, it is likely that as p-tau deposition becomes more severe, neuronal death and dysfunction will become more related to pathogenic protein accumulation.

One limitation of our study is the small amount of tissue in each sample. CTE is an inherently patchy disease, which is diagnosed on the basis of the presence of a pathognomonic CTE lesion consisting of a focus of perivascular p-tau accumulation at the depth of the cortical sulcus. It is therefore possible that sampling may have missed regions of important cellular responses. Future studies of RHI-exposed individuals should aim to sample from multiple areas of the brain to improve detection of cellular responses. Similarly, owing to the limited availability of donated samples from individuals under the age of 50, the current sample size is not as large as those in other recent snRNA-seq studies that might have 100 or more cases. However, as long as expression differences are robust and consistent, as observed in this study, disease-specific effects can be identified with smaller sample sizes^43^. However, follow-up studies to increase the sample size could help identify additional cellular heterogeneity and enhance the resolution of more subtle changes. Finally, owing to the inherent difficulties in acquiring non-disease, non-RHI-exposed post-mortem samples from young humans, some control cases included in the Nissl quantification were from female donors, which may complicate direct comparisons with samples from male athletes. However, we found no statistical correlation between sex and neuronal density.

These results highlight the growing concerns linked to long-term RHI exposure from contact sports. The data presented here provide direct evidence demonstrating that RHI-driven cellular perturbations occur prior to the development of CTE and can be observed in young individuals, many of whom exhibit no obvious brain pathology. Novel biomarkers and therapeutic interventions will be vital in identifying the early changes observed in contact sport athletes prior to developing neurodegeneration.

## Methods

### Neuropathological Diagnosis

Brain tissue was obtained from the CTE and the National Center for PTSD Brain Banks. Identical intake and tissue processing procedures occur with both brain banks. Four controls included in Nissl quantification were provided by the Iowa Neuropathology Resource Laboratory. Neuropathological examination was performed by board certified neuropathologists as described previously^10,44^. Diagnosis of CTE was determined using published consensus criteria^10,44^. Demographics such as athletic history, military history, traumatic brain injury history, and RHI history were queried during telephone interview with next of kin as detailed previously^10,44^. Institutional review board approval for brain donation and informed consent for research was obtained through the Boston University Alzheimer’s Disease and CTE Center, Human Subjects Institutional Review Board of the Boston University School of Medicine, and VA Boston Healthcare System (Boston, MA). Individuals were included in the snRNA-seq and single-molecule fluorescence in situ hybridization (smFISH) experiments based on frozen tissue availability, quality (RNA integrity number (RIN) > 4) and diagnosis. Those used for immunohistochemistry were included based on the same criteria except frozen tissue availability and RIN. Exclusion criteria included neuropathological diagnosis other than CTE, moderate to severe traumatic brain injury directly prior to death, age of death greater than 51 or less than 25. Control cases did not have exposure to any RHI, were negative for any neurodegenerative disease, and did not carry any diagnosis of a neuropsychological disorder.

### snRNA-seq

Fresh frozen brain tissue was collected from the dorsolateral frontal cortex of each donor at the depth of the cortical sulcus. Visual delineation of grey and white matter was used to collect 50 mg of grey matter tissue. Tissue was processed and cleaned of white matter prior to homogenization at two levels. First, when removing samples from frozen coronal slabs, the unbiased technician visually inspected and avoided white matter that could be adjacent to target grey matter. Second, immediately before tissue homogenization, a second technician inspects the tissue and removes any remaining white matter. This preparation allows for a highly specific grey matter enrichment. Nuclei isolation and sorting were performed on two donor samples per day, randomizing for diagnosis and age. Tissue was kept on ice throughout nuclei isolation. Tissue was homogenized and lysed in NST Buffer with DAPI (146 mM NaCl, 10 mM Tris, 1 mM CaCl_2_, 21 mM MgCl_2_, 0.1% BSA, 0.1% NP-40, 40 U ml^−1^ Protector RNase Inhibitor and DAPI) and snipped with scissors on ice for 10 min. Debris was removed using a 70-μm filter. Cells were spun down and resuspended in nuclei storage buffer (2% BSA, 400 U ml^−1^ Protector RNase Inhibitor) to reach a concentration of 500–1,000 nuclei per μl. Nuclei were purified for DAPI-positive cells with a FACS Aria flow cytometer to remove debris and processed using the Chromium Next GEM Single Cell 3′ Reagents Kit V2 (10x Genomics) to create cDNA libraries. Samples were pooled in two batches sequenced with Azenta to a read depth of 30,000 reads per cell on an Illumina NovaSeq.

### Processing, quality control and clustering of snRNA-seq data

CellRanger v.6.0.1 was used to align reads to the GRCH38 reference and generate filtered count matrices containing 233,555 cells across all samples. The runCellQC function in the singleCellTK R package was used to generate quality control metrics and doublet calls^45,46^. Contamination from ambient RNA was identified using decontx using the full raw matrix as the ‘background’ for each sample^47^. Nuclei were removed if they had ambient RNA contamination fraction greater than 0.3, mitochondrial or ribosomal percentage greater than 5%, total counts less than 750, or genes detected less than 500. The data were not down sampled to maximize capture of rare populations. The Seurat workflow within the singleCellTK package was used for clustering starting with the decontaminated counts from decontx^48^. In brief, the data were normalized and scaled using runSeuratNormalizeData and runSeuratScaleData. Highly variable genes were identified using runSeuratFindHVG with the method vst. Principal components were determined using runSeuratPCA. UMAP dimensionality reduction was calculated using runSeuratUMAP. Clusters across all cell types were identified using the runSeuratFindClusters function at a resolution of 0.3. After initial clustering all the cells, clusters that were predominantly doublets (>50%) were removed and produced the final dataset of 170,717 nuclei (Extended Data Fig. 1h–k). Associations with post-mortem interval (PMI), age at death and sequencing batch were performed using Pearson’s correlation analysis in R (Supplementary Fig. 4). Age at death was associated with only excitatory neuron L5_FEZF2_PCP4_RPRM and inhibitory neuron PVALB_SCUBE_PTPRK proportions. Therefore, age was not included in regressions performed with sequencing data. PMI correlated with only one microglial subtype (RHIM1), perivascular macrophages, an excitatory neuron subtype (L2_4CUX2_COL5A2) and several oligodendrocyte subtypes. Sequencing batch was associated with one cluster of OPCs and was therefore not included in analyses.

All GO analysis was performed using MetaScape default settings^49^. DEG lists for all comparisons available in Supplementary Tables 6–16.

### Cell-type identification

Cell-type markers verified by previous human snRNA-seq studies were used to identify clusters that belonged to individual cell types (Extended Data Fig. 1m,n). Cell types were subsetted out using subsetSCEColData and reclustered by the same Seurat method described above with the addition of running Harmony to account for sample-to-sample variability^50^. Clusters expressing high levels of >1 cell-type marker were removed. Excitatory and inhibitory neurons identified from the full dataset were clustered together to determine neuronal subtypes. Four clusters (1, 2, 19 and 21) were found to express low levels of neuronal genes and astrocytic genes (*SLC1A2* and *SLC1A3*), and were single-batch enriched (80–90%) therefore these clusters were not included in downstream analysis (Extended Data Fig. 8a–d).

### Module analysis

#### Celda

Gene co-expression modules were identified using Celda^51^. Celda utilized Bayesian hierarchical linear mixed effects models to identify modules of genes that are expressed together. A workflow overview can be found in Extended Data Fig. 4. Celda was run on cellular subtypes to determine module scores on a cell-wise basis and plotted across cellular subtypes. Statistical analysis of module enrichment was performed using a linear mixed effects model using sample ID as a covariate. For microglia, cell subtypes were compared to homeostatic microglia as a baseline, for endothelial cells Cap1 was used, for astrocytes Astro1 (homeostatic astrocytes) were used as a baseline. Celda module analysis was plotted as Violin plots as these types of plots demonstrate statistical differences and also allow for visualization of the variance within the data (Supplementary Figs. 1, 7, 8). Additionally, to help further validate findings, radar plots for each Celda module were also provided to help visualize module distribution among all groups (Supplementary Figs. 3, 5 and 10).

#### hdWGCNA

hdWGCNA (v.0.4.5) was also run to validate gene co-expression modules in astrocytes, microglia, and endothelial cells. The hdWGCNA workflow was run with default parameters except min_cells was set to 25 and *k* was set to 10 for the metacells analysis performed by the MetacellsByGroups function. Additionally, minModuleSize was set to 25 in the ConstructNetwork function for astrocytes and microglia. Radar plots were provided to demonstrate cell-type distribution. Metascape^49^ was used to generate GO analyses for Fig. 2f. Statistics for GO were generated with GSEA and single-tailed hypergeometric test with Benjamini–Hochberg multiple hypothesis correction.

hdWGCNA and Celda Modules were compared against each other for further validation. All major modules of interest could be observed in both module analyses (Supplementary Figs. 2c, 6d and 9d). The discrepancy between module numbers with hdWGCNA and Celda was the result of how each technique process data. Celda clusters every gene into a module, in contrast to hdWGCNA that does not. Celda also captures modules that are broadly expressed across many clusters rather than modules only expressed in small numbers of clusters. Biological function of each module was assessed with the EnrichR package to validate functional significance. Finally, in order to efficiently run hdWCGNA on single cell data, a prior step must be performed that reduces the cells to ‘metacells’. According the hdWGCNA tutorial, “metacell aggregation approach does not yield good results for extremely underrepresented cell types”, which probably also contributes to the reduced module number. Although module numbers may differ, important modules of interest were preserved through both datasets.

All module genes and statistical analysis can be viewed in Supplementary Tables 17–19, analysis code is available on GitHub at www.github.com/morganebutler/singleCellScripts.

### External dataset comparison

The Sun et al. dataset^23^ was downloaded from https://compbio.mit.edu/microglia_states/. Another Sun et al.^25^ dataset was downloaded at http://compbio.mit.edu/scADbbb/. For the microglia, bootstrapping was performed by randomly sampling 80% of the Sun dataset with replacement for 50 iterations. For each iteration, FindTransferAnchors from the Seurat package was used to project the current microglia dataset onto the Sun UMAP space, and MapQuery to predict microglia labels. Label calls were recorded for each iteration and the label consistency was reported as the percentage of iterations the same label was called in Extended Data Fig. 5d,e.

For Visium projection of astrocyte subtype genes, publicly available Visium data from human cortex (Adult Human Brain 1) were downloaded from the 10x Genomics website. The Seurat function AddModuleScore was used to create a per-spot score for astrocyte subtype gene expression (significantly upregulated genes in each subtype). Plots were created with SpatialFeaturePlot and displayed in Extended Data Fig. 6i.

### MultiNicheNet

Ligand–receptor pair analysis was performed using MultiNicheNet, an adaptation of nichenet that allows for comparison across more than two condition groups. In brief, this method uses known datasets of ligand–receptor pairs and their downstream targets to identify potentially upregulated cell signalling pathways across cell types accounting for differential expression of genes across groups. MultiNicheNet also uses prioritization of top ligand–receptor pairs to help identify signalling pathways of interest. Contrasts for differential gene expression were set as RHI versus control, and CTE versus RHI to determine RHI and CTE-specific signalling pathways. Finalized cell-type objects were combined and run through the MultiNicheNet pipeline with the exclusion of T cells due to low cell numbers. Analysis was performed without alteration to publicly available code, save for the contrasts used.

### Histological tissue processing

Formalin-fixed, paraffin-embedded tissue was sectioned and labelled as described^52^. In brief, 10-μm sections were allowed to dry, baked, dewaxed and rehydrated prior to antibody labelling. For immunofluorescent staining, epitope retrieval was performed using a pH 6 or pH 9 buffer and boiling for 15 min in the microwave. Sections were blocked for 30 min at room temperature with 3% donkey serum and primary antibodies (Supplementary Table 4) were conjugated for 1 h at room temperature. Secondary antibodies were conjugated for 30 min, and Opal TSA dyes were incubated for 10 min. Slides were coverslipped with ProLong Gold Antifade mounting medium (Invitrogen) and imaged at 20× or 40× on a Vectra Polaris whole-slide scanner with the appropriate filters. Images were spectrally unmixed using inForm software prior to image analysis. For Nissl staining, sections were hydrated and stained in 0.01% thionin for 20–40 s and dehydrated back to xylene before coverslipping in Permount mounting media and imaging on an Aperio GT450 scanner at 40×. As formalin-fixed histologic tissue was more readily available than frozen samples, more samples could be utilized for immunohistochemistry and in situ hybridization experiments. A full list of samples that were included in each immunohistochemistry experiment is shown in Supplementary Tables 2 and 3.

### smFISH and immunohistochemistry codetection

Tissue was embedded in Optimal Cutting Temperature medium (Sakura Tissue-Tek) and was brought to cryostat temperature (−20 °C) before cutting. Chuck temperature was raised to −12°/−10 °C for optimal cutting conditions. Tissue was sectioned at 16 µm thickness onto Fisher SuperFrost slides. Direction of tissue orientation relative to the depth of the cortical sulcus was randomized across samples. Sections were fixed in cold 4 °C 10% neutral buffered formalin for 60 min and dehydrated in 50%, 70%, 100% and 100% ethanol for 5 min each at room temperature. Fluorescent in situ hybridization was performed using RNAScope kits (Advanced Cell Diagnostics) (Supplementary Table 5) optimized on the Leica BOND Rx automated slide staining system. Slides were pretreated with protease for 15 min. Opal TSA dyes were used for visualization at a concentration of 1:300–1:500. A positive and negative control probe was run for each block before staining with targeted probes. For immunohistochemical codetection of p-tau and GLUT1, sections were run through the RNAScope protocol as described and then manually stained with the AT8 or GLUT1 antibody (Supplementary Table 4) with the immunohistochemical protocol described in ‘Histological analysis’ without the antigen retrieval. Samples included in each smFISH experiment are listed in Supplementary Table 2. Not all samples were used across every smFISH experiment due to exhaustion of sample blocks.

### Image analysis

Analysis of fluorescent RNAScope fluorescence in situ hybridization (FISH) was performed in Indica Labs HALO using the FISH v.3.2.3 algorithm or the FISH-IF v.2.2.5 algorithm. Thresholds for FISH probe positivity for was set manually for each probe (*HIF1A*, *SPP1*, *P2RY12*, *ITGAV*, *TGFB1*, *TGFBR2*, *LAMP5* and *CUX2*) and kept consistent across samples. It should be noted that SPP1 is not exclusively expressed by microglia, and DEG analysis demonstrated that only oligodendrocytes showed elevated expression of *SPP1* in our dataset (Supplementary Table 6b). However, colocalization with microglia markers allows for a microglia-specific count of *SPP1* activity. Gene expression was determined by the ‘probe cell intensity’ in HALO because this measure is agnostic to manual single copy intensity settings. The background on GLUT1 staining in FISH sections was variable due to protease treatment from RNAScope and thresholds were manually adjusted to remove background staining. Vessel proximity analysis was performed by evaluating *TGFB1*+*P2RY12*+ cells and GLUT1^+^*ITGAV*+*TGFBR2*+ cells and using the ‘proximity analysis’ algorithm in the HALO spatial analysis settings. The number of unique marker-positive microglia/vessel pairs within 25 µm were evaluated. Density heat maps for *CUX2*+*LAMP5*+ staining were created using the ‘density heatmap’ function within HALO spatial analysis. Depiction of how the sulcus and crest were annotated can be found in Extended Data Fig. 10d. To validate consistency between image analyses methods and snRNA-seq results, seven samples that were included in both RNAScope and snRNA-seq methods were compared and cellular proportions of *CUX2*+*LAMP5*+ neurons significantly correlated (*P* = 0.02; Extended Data Fig. 10c).

Analysis of immunohistochemistry protein staining was performed using the HALO Object Colocalization v.2.1.4 and HighPlex v.4.3.2 algorithm. Microglial P2RY12 was assessed by DAPI^+^IBA1^+^ nuclei and P2RY12^hi^^/low^ thresholds were set manually. High P2RY12 was defined as having at least 70% of the nucleus stained, low P2RY12 was defined as less than 70% of the nucleus stained as demonstrated visually in Fig. 2n. Only 5.4% of all IBA1^+^ or P2RY12^+^ cells were P2RY12^+^IBA1^−^, suggesting that 94.6% of labelled microglia were assessed. IBA1^+^P2RY12^−^ cells may have been captured in our P2RY12^low^ categorization, however previous studies have shown that these cells are low in abundance and likely represent infiltrating macrophages which have been shown to be present mainly at lesioned vessels in CTE which are also sparse in our cohort^53,54^.

Analysis of Nissl staining was performed using the HALO Nuclei Segmentation AI algorithm. Neurons were selected for training based on previously published criteria^55^. In brief, the classifier was given examples of brain parenchyma annotated for neurons which were considered cells with a Nissl-positive cytoplasm and a visible nucleus (Extended Data Fig. 9h). Nissl+ densities across batches were not significantly different and statistical tests of Nissl densities were corrected for staining batch. For FISH and Nissl sections, the depth of the cortical sulcus was defined and annotated as the bottom third of a gyral crest and sulcus pair. Layer 2/3 and layers 4–6 were annotated using layer-specific FISH markers or for Nissl by an expert observer.

### Software and code

The following code and software was used for the analyses: CellRanger v.6.0.1 was used to align reads to the GRCH38 reference and generate filtered count matrices. All other analyses were performed in R v.4.2.1 and Python v.3.10.12 using standard functions unless otherwise stated. Specific versions of packages used are listed in available GitHub code. The following packages were used: CellRanger v.6.0.1, singleCellTK v.2.8.0, Seurat v.4.3.0, scater v.1.24.0, harmony v.0.1.1, RColorBrewer v.1.1.3, ComplexHeatmap v.2.14.0, ArchR v.1.0.2, muscat v.1.12.1, readr v.2.1.4, ggplot2 v.3.4.2, ggsignif v.0.6.4, ggpubr v.0.6.0, magrittr v.2.0.3, scCoda v.0.1.9 Python package, celda v.1.19.1 and hdWGCNA v.0.4.5.

HALO v.3.6.4134.193, HALO AI v.3.6.4134, HALO Object Colocalization v.2.1.4 algorithm and FISH v.3.2.3 algorithm were used to analyse the histological and Nissl images. InForm v.2.5.1 was used to spectrally unmix fluorescent in situ hybridization images.

### Inclusion and ethics statement

The research has included local researchers through the research process and is locally relevant with collaborators. All roles and responsibilities were agreed amongst collaborators ahead of the research. The research was not severely restricted in the setting of researchers. The study was approved by the Institutional review board through the Boston University Alzheimer’s Disease and CTE Center, Human Subjects Institutional Review Board of the Boston University School of Medicine, VA Bedford Healthcare System, VA Boston Healthcare System, and Iowa Neuropathology Resource Laboratory. The research did not result in stigmatization, incrimination, discrimination, or risk to donors or research staff. No materials have been transferred out of the country. Local and regional research relevant to the study has been included in the citations.

### Statistics and reproducibility

Analyses were performed using GraphPad Prism 10, SPSS v.29 and R (v.4.2.1) packages ggsignif, muscat, scater, and the Python (v.3.10.12) package scCoda. Dirichlet multinomial regression was used to test for cell type and excitatory neuron cell-type enrichment using the scCoda v.0.1.9 Python package^13^. Celda module expression was evaluated using linear mixed effects modelling, accounting for individual sample differences. Comparisons of cell-type proportions across the three pathological groups were performed using ANOVA with Bonferroni correction, Brown Forsyth with Dunnett post-hoc test, or chi-squared test as indicated in figure legends. Comparison across control and RHI-exposed groups was performed with a *t*-test with Welch correction or Mann–Whitney *U-*test, as indicated in the figure legends. Bar plots denote error with s.e.m. Scatter plots denote error with a grey outline of the 95% confidence interval. Evaluation of in situ hybridization analysis was performed using linear regression. P-tau burden was normalized using log_10_ transformation of positive area density. Nissl^+^ neuron count comparisons to years of exposure were assessed using linear regression and correcting for age at death and staining batch. Jaccard similarity scoring was performed using the GeneOverlap package by comparing lists of DEGs. All DEGs were filtered by a log_2_-transformed fold change of 0.15 and false discovery rate (FDR) of <0.05. Chi-squared tests for cellular abundance were performed using the base R chisq.tst function. GO analysis *P* values were acquired through MetaScape analysis. GO statistics were calculated with GSEA and single-tailed hypergeometric test with Benjamini–Hochberg multiple hypothesis correction. Years of football play was used as a variable for exposure throughout the text instead of total years of play (which includes exposure from all sports) played because it was a more consistent predictor of cellular changes.

snRNA-seq tissue isolation was performed once per each individual. Reproducibility was assessed through comparison to other published datasets^23,25^. As detailed in Extended Data Figs. 3, 5 and 7, there was significant overlap between our subtypes and other previously published subtypes, highlighting that our results are highly reproducible. For all histological antibody, Nissl and in situ hybridization staining, individual cases were stained and analysed once per each experiment. Histologic methods were validated and optimized prior to the start of the experiment to ensure proper labelling and accurate downstream analyses as discussed in the previous sections.

### Reporting summary

Further information on research design is available in the Nature Portfolio Reporting Summary linked to this article.

## Online content

Any methods, additional references, Nature Portfolio reporting summaries, source data, extended data, supplementary information, acknowledgements, peer review information; details of author contributions and competing interests; and statements of data and code availability are available at 10.1038/s41586-025-09534-6.

## Supplementary information

Supplementary Information This file contains Supplementary Figs. 1–10

Reporting Summary

Supplementary Tables Supplementary Tables 1–19

## Data Availability

Code can be found at www.github.com/morganebutler/singleCellScripts.

## Data availability

Data are available at GEO accession number GSE261807. All other data supporting the findings of this study and unprocessed images are available upon request. Request for tissue or digital images can be made by emailing the corresponding author or through the Boston University ADRC request portal at https://wwwru.bumc.bu.edu/BUADC/RequestBrainTissue.aspx. The following public databases were used for the study: GRCH38 reference GCF_000001405.26, Sun et al. microglia dataset (https://compbio.mit.edu/microglia_states/), Visium spatial expression (https://www.10xgenomics.com/datasets/adult-human-brain-1-cerebral-cortex-unknown-orientation-stains-anti-gfap-anti-nfh-1-standard-1-1-0) and Sun et al. vascular dataset (http://compbio.mit.edu/scADbbb/).
