# Supplementary material for: Repeated head trauma causes neuron loss and inflammation in young athletes
Source: Nature. 2025 Sep 17;647(8088):228–37. doi: 10.1038/s41586-025-09534-6 (PMC12589125; doi:10.1038/s41586-025-09534-6)
Supplement: Supplementary file 1 — This file contains Supplementary Figs. 1–10 [file 41586_2025_9534_MOESM1_ESM.pdf]

nature portfolio
https://doi.org/10.1038/s41586-025-09534-6

Supplementary information

Repeated head trauma causes neuron loss and inflammation in young athletes

In the format provided by the authors and unedited

Supplementary Figure 1

Microglia Modules L1- L90

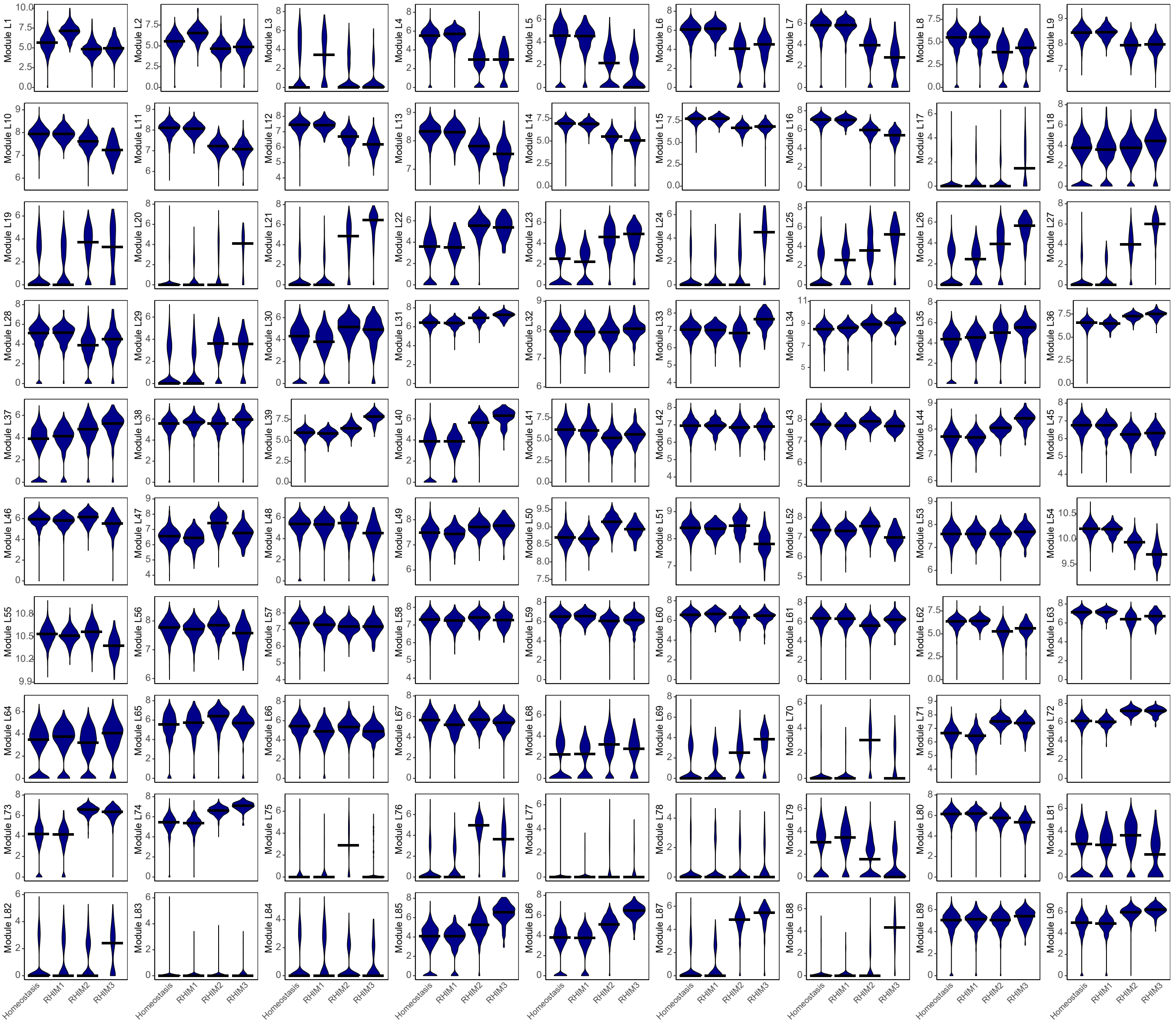

Supplementary Figure 1. Microglia Celda Modules. Violin plots depicting Celda module expression for modules 1-90 across Homeostatic, RHIM1, RHIM2, and RHIM3 microglial clusters. Black bar is the median statistic from ggsignif. n = 28 individuals.

Supplementary Figure 2

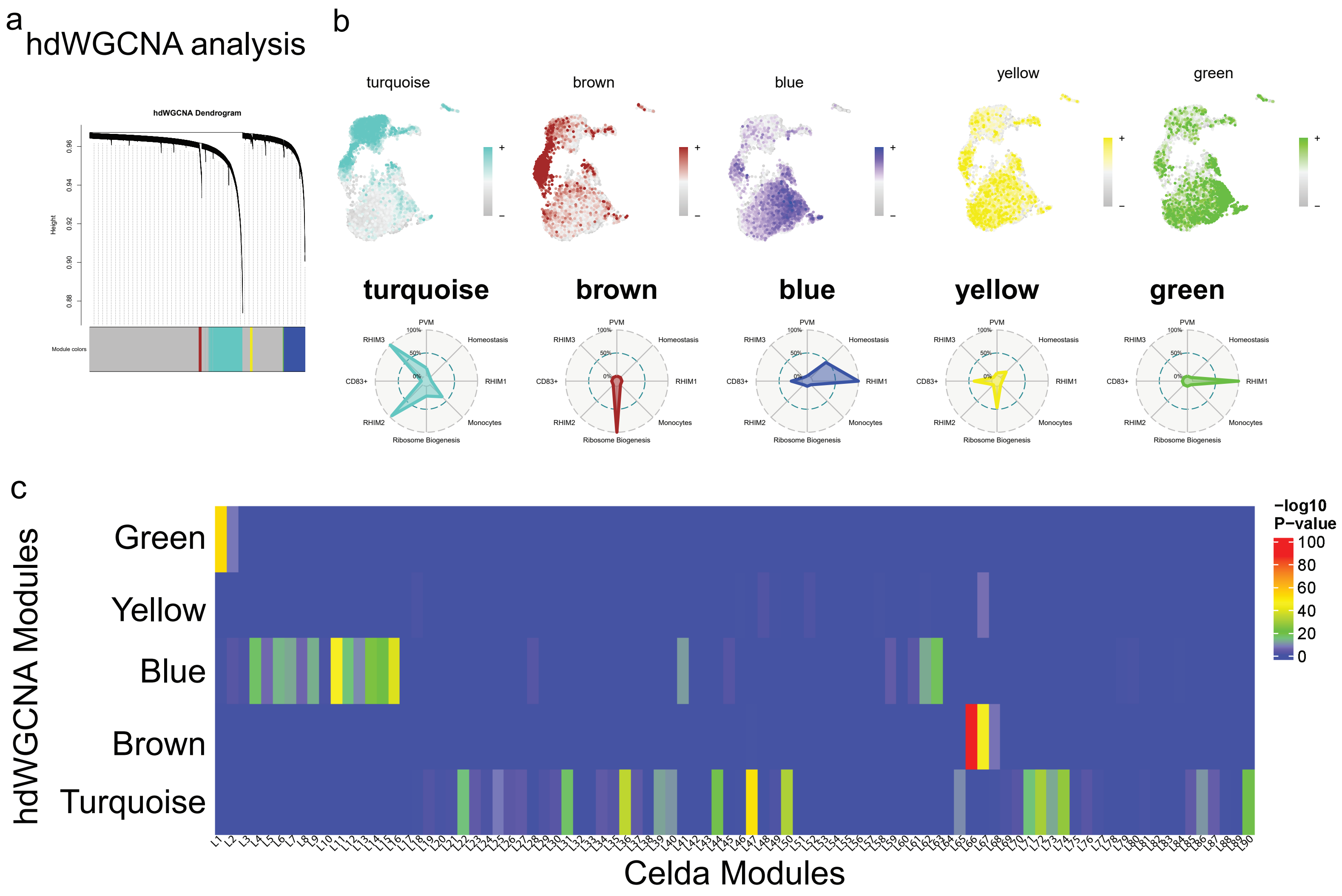

Supplementary Figure 2. Microglia hdWGCNA. a. hdWGCNA dendrogram to visualize difference in coexpression modules. Each leaf represents a single gene. Module assignments are indicated by color. **b**. Projection of each module onto microglia UMAP. Corresponding radar plots to demonstrate cell type cluster associations are located below each respective UMAP. **c**. Jaccard similarity score comparing hdWGCNA derived gene modules to Celda derived gene modules. As validation, all major modules of interest could be observed in both module analyses. Color gradient denotes -log10 p-value significance. n = 28 individuals.

Supplementary Figure 3

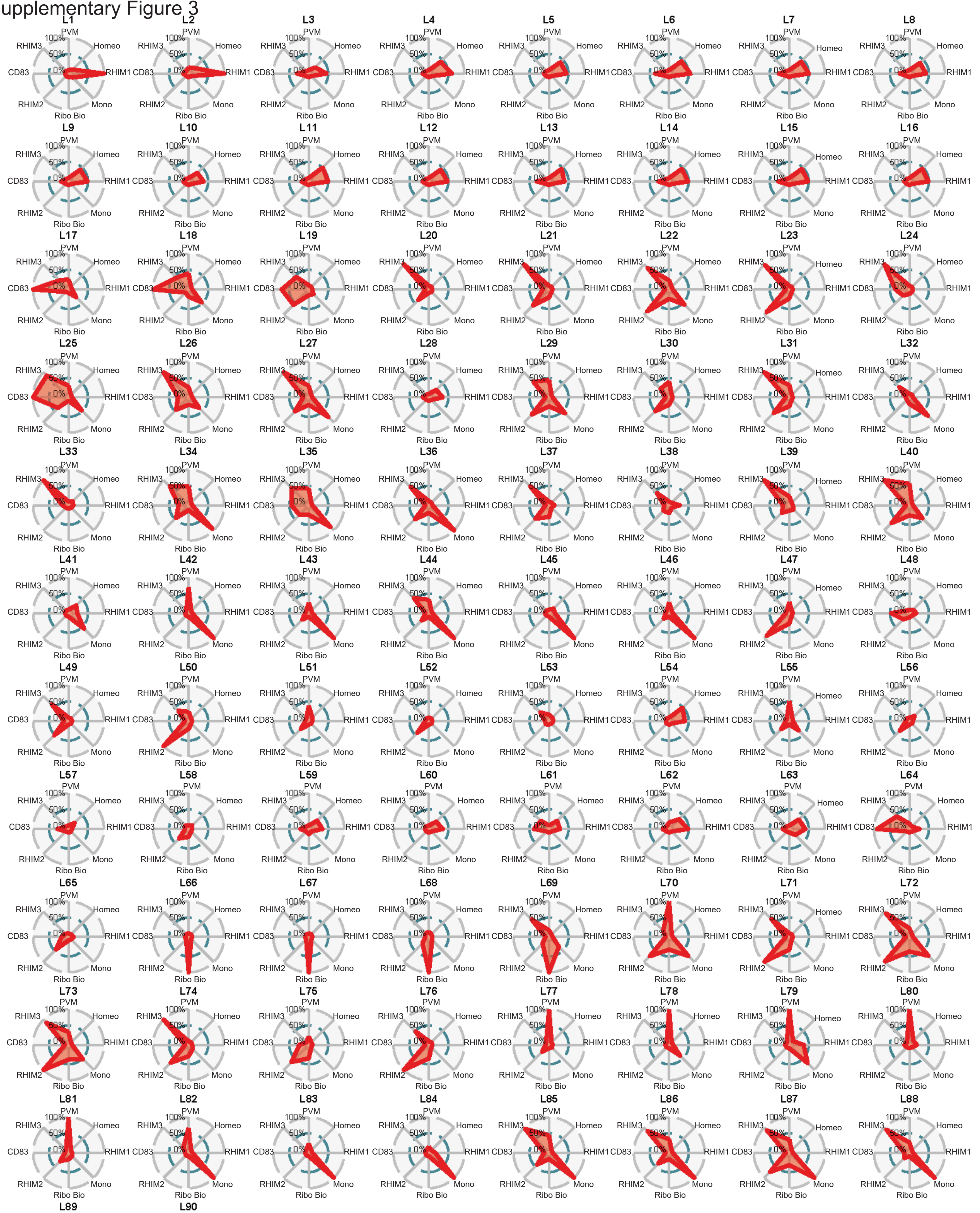

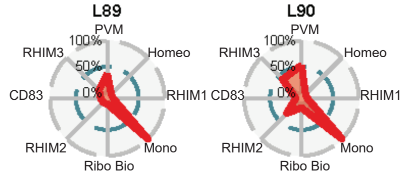

Supplementary Figure 3. Microglia Celda Radar Plots. Radar plots generated from Celda modules (Supplementary table 17a) to visualize module expression in microglia subpopulations. n = 28 individuals.

Supplementary Figure 4

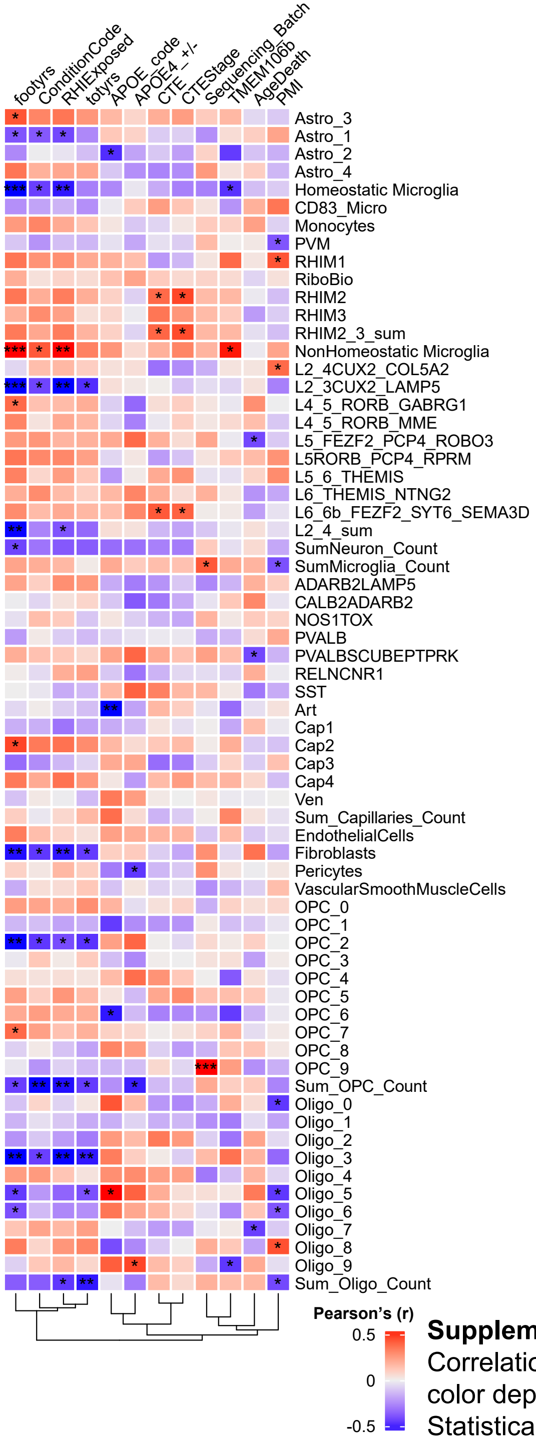

Supplementary Figure 4. Cellular subtype metadata correlations. a. Correlation heatmap of selected metadata and cellular subtypes. Heatmap color depicts and direction and magnitude of Pearson's r correlation value. Statistical analysis performed by pearson correlation. *, p <0.05, **, p < 0.01, ***, p < 0.001. n = 28 individuals.

Supplementary Figure 5

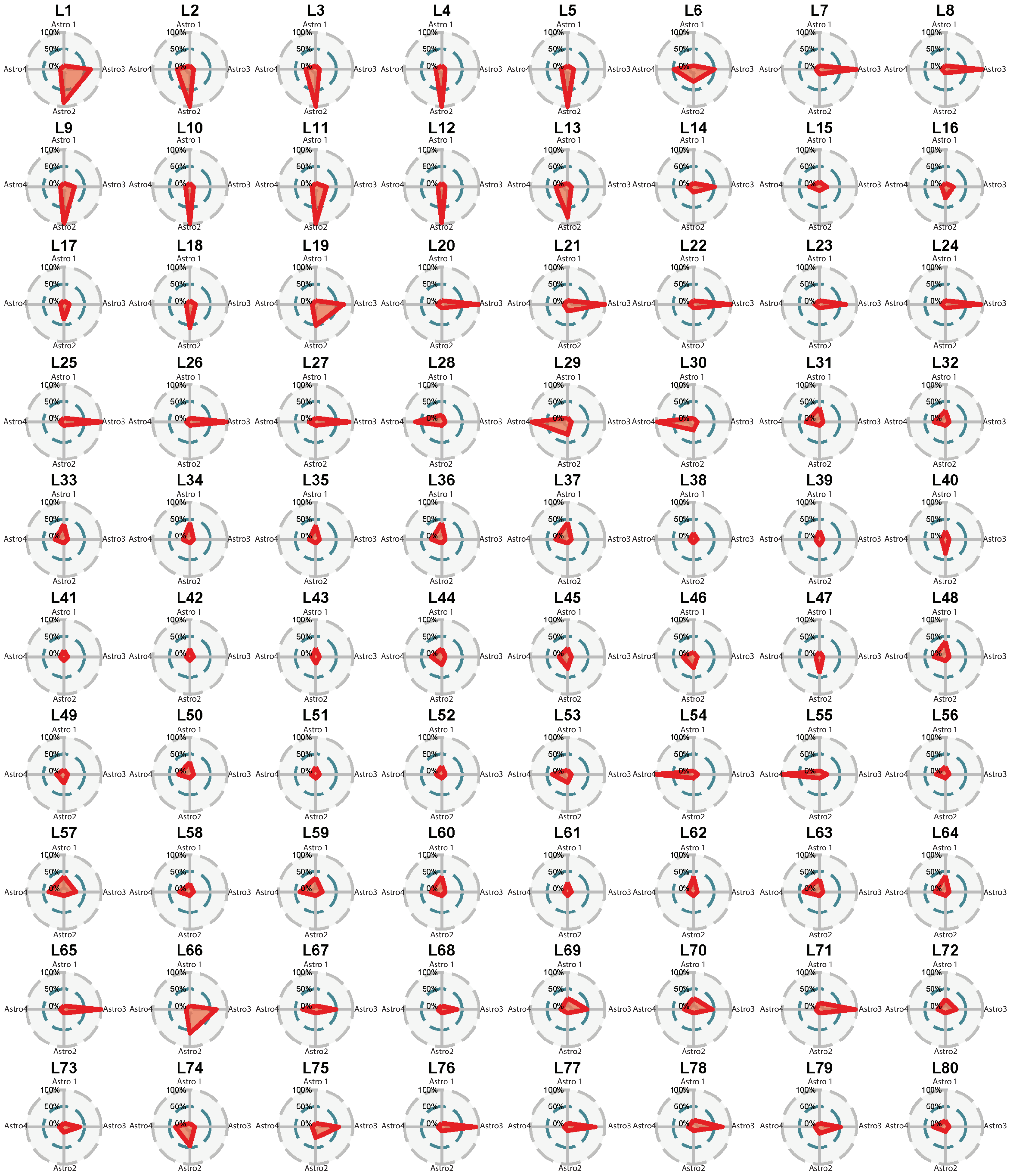

Supplementary Figure 5. Astrocyte Celda Radar Plots. Radar plots generated from Celda modules (Supplementary Table 19a) to visualize module expression in astrocyte subpopulations. n = 28 individuals.

Supplementary Figure 6

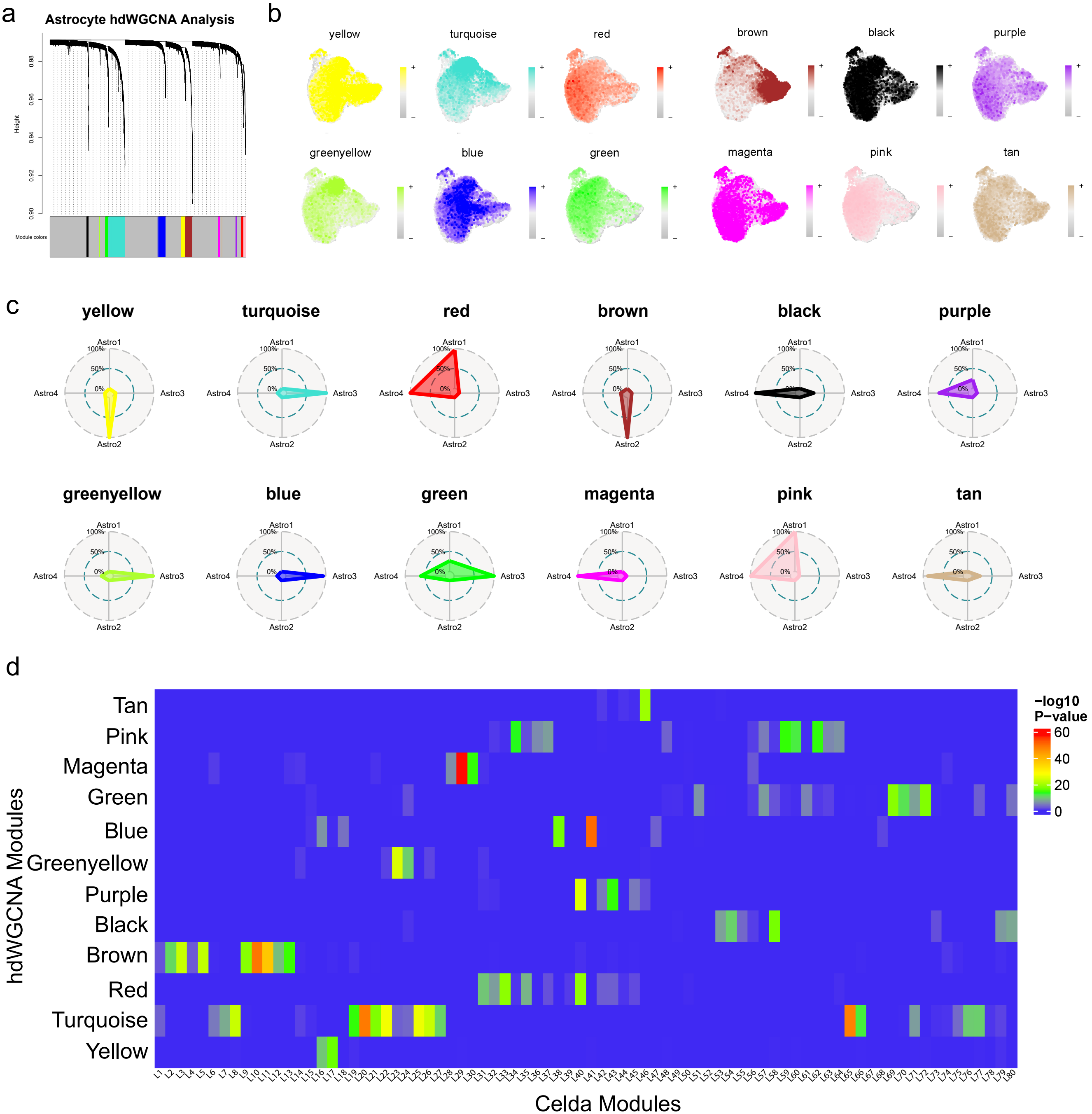

Supplementary Figure 6. Astrocyte hdWGCNA Modules. a. hdWGCNA dendrogram to visualize difference in coexpression modules. Each leaf represents a single gene. Module assignments are indicated by color. b. Projection of each module onto astrocyte UMAP. c. Corresponding radar plots to demonstrate cell type cluster associations for each module. c. Jaccard similarity score comparing hdWGCNA derived gene modules to Celda derived gene modules. As validation, all major modules of interest could be observed in both module analyses. Color gradient denotes -log10 p-value significance. n = 28 individuals.

Supplementary Figure 7
Astrocyte Modules L1-L80

Supplementary Figure 7. Astrocyte Celda Modules. Violin plots depicting Celda module expression for modules 1-80 across Astro1, Astro2, Astro3, and Astro4 astrocyte clusters. Black bar is the median statistic from ggsignif. n = 28 individuals.

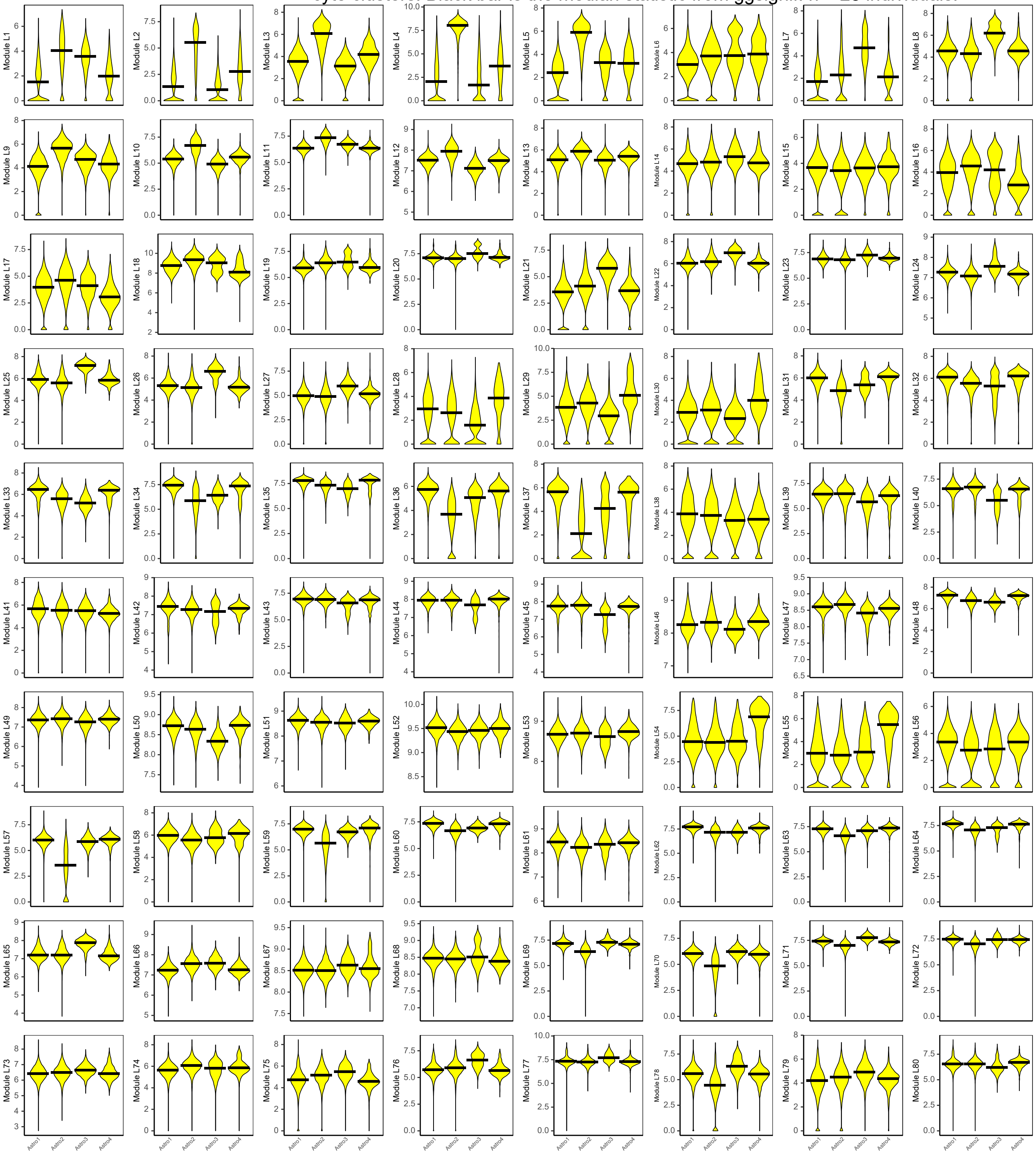

Supplementary Figure 8

Endothelial Cell Modules L1- L60

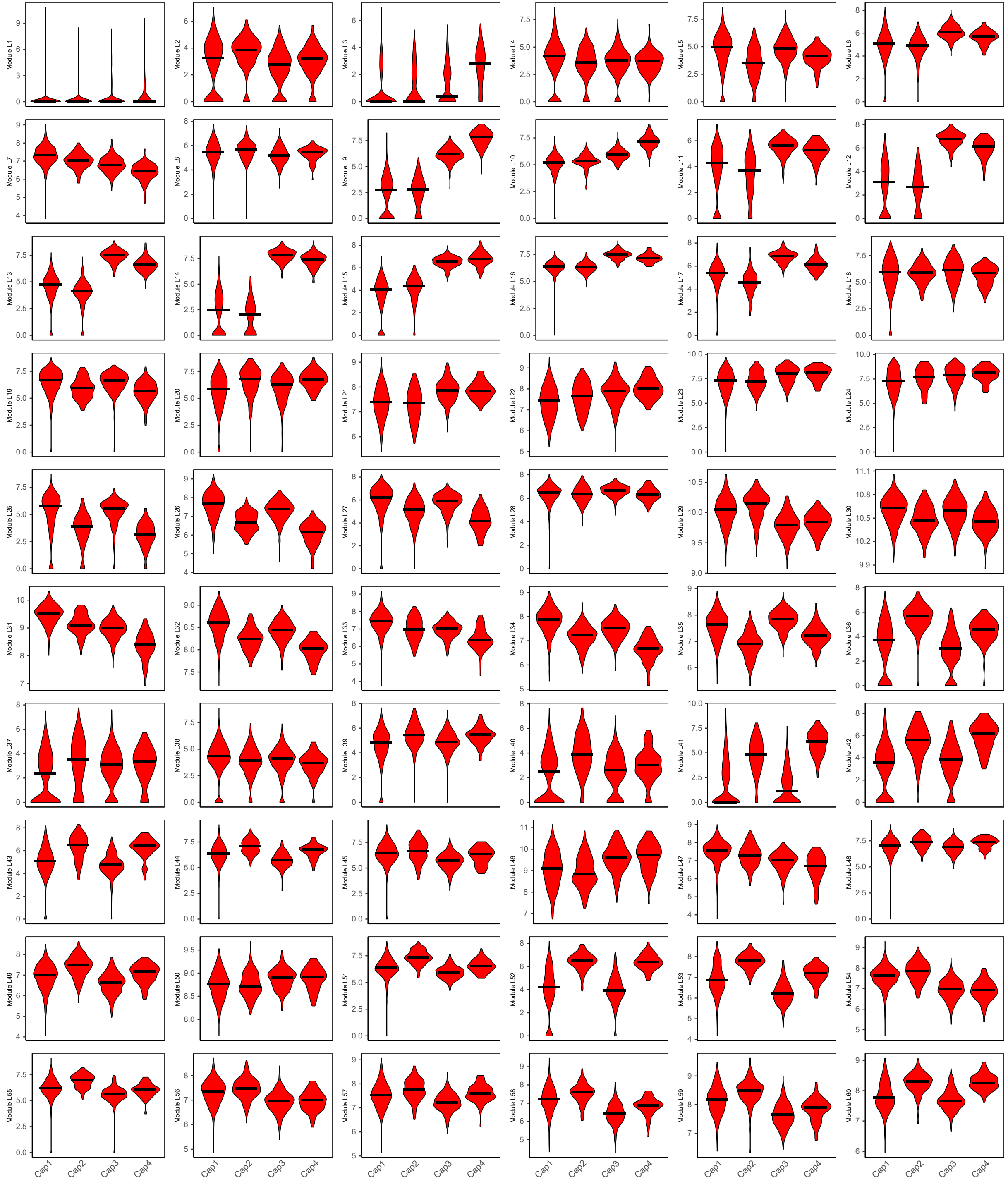

Supplementary Figure 8. Endothelial Cell Celda Modules. Violin plots depicting Celda module expression for modules 1-60 across Cap1, Cap2, Cap3, and Cap4 endothelial cell clusters. Black bar is the median statistic from ggsignif. n = 28 individuals.

Supplementary Figure 9

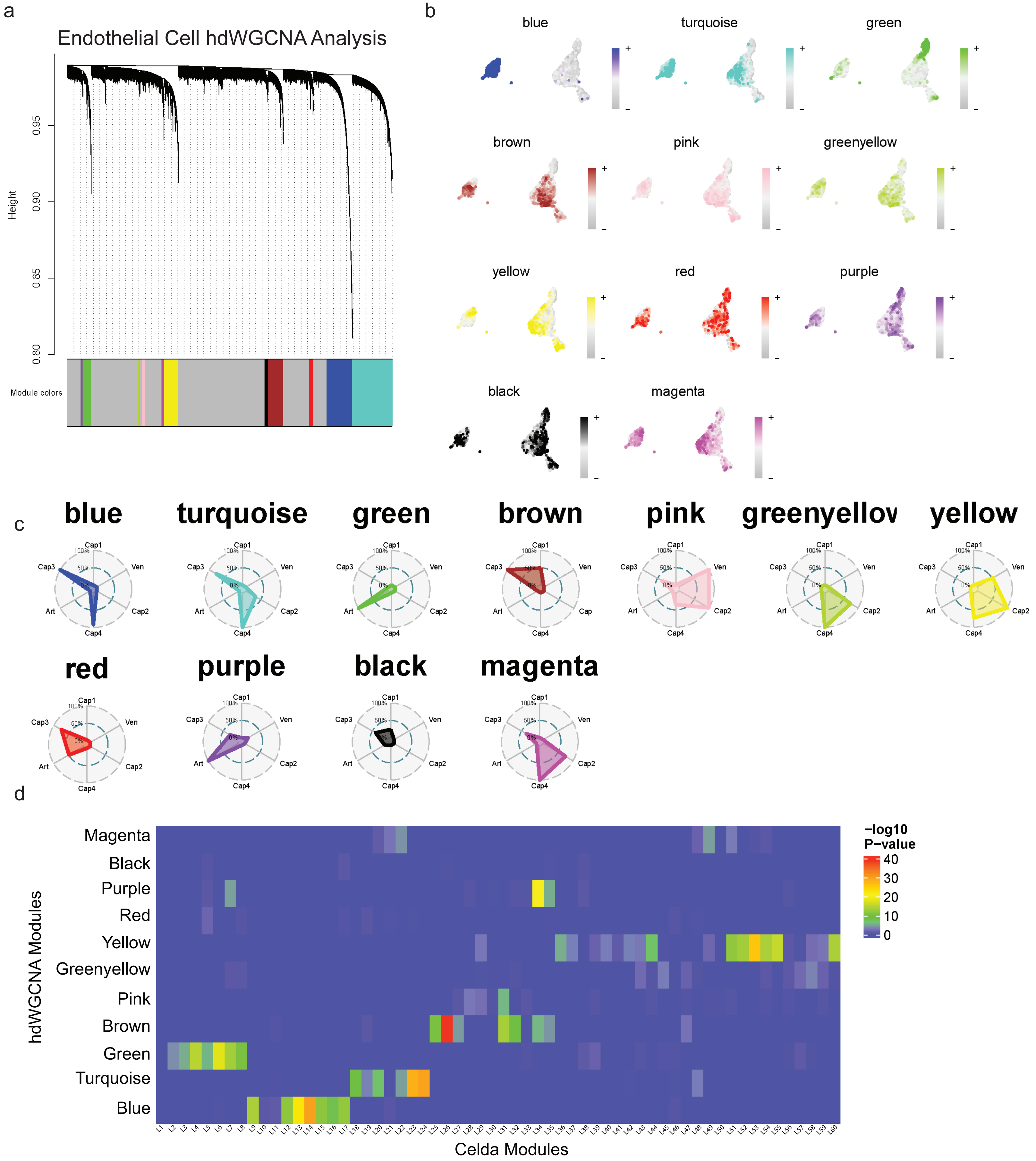

Supplementary Figure 9. Endothelial hdWGCNA Modules. a. hdWGCNA dendrogram to visualize difference in coexpression modules. Each leaf represents a single gene. Module assignments are indicated by color. b. Projection of each module onto endothelial cell UMAP. c. Corresponding radar plots to demonstrate cell type cluster associations for each module. c. Jaccard similarity score comparing hdWGCNA derived gene modules to Celda derived gene modules. As validation, all major modules of interest could be observed in both module analyses. Color gradient denotes -log10 p-value significance. n = 28 individuals.

Supplementary Figure 10

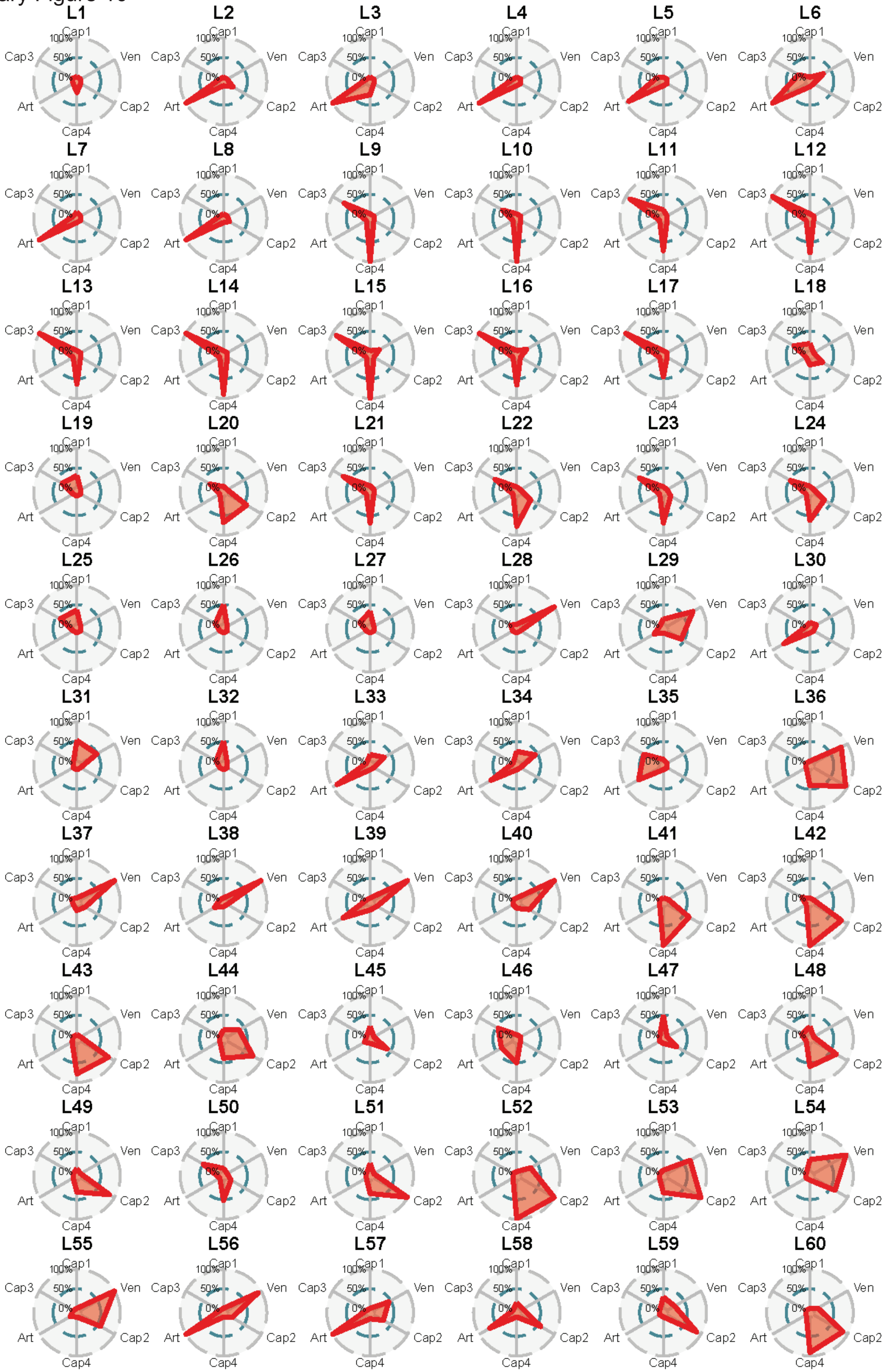

Supplementary Figure 10. Endothelial Cell Celda Radar Plots. Radar plots generated from Celda modules (Supplementary Table 18a) to visualize module expression in endothelial cell subpopulations. n = 28 individuals.
