# Supplementary material for: Repeated head trauma causes neuron loss and inflammation in young athletes
Source: Nature. 2025 Sep 17;647(8088):228–37. doi: 10.1038/s41586-025-09534-6 (PMC12589125; doi:10.1038/s41586-025-09534-6)
Supplement: Supplementary file 2 — Reporting Summary [file 41586_2025_9534_MOESM2_ESM.pdf]

nature portfolio

Corresponding author(s): Jonathan Cherry

Last updated by author(s): Jul 28, 2025

Reporting Summary

Nature Portfolio wishes to improve the reproducibility of the work that we publish. This form provides structure for consistency and transparency in reporting. For further information on Nature Portfolio policies, see our Editorial Policies and the Editorial Policy Checklist.

Statistics

For all statistical analyses, confirm that the following items are present in the figure legend, table legend, main text, or Methods section.

n/a	Confirmed	
☐	☒	The exact sample size (*n*) for each experimental group/condition, given as a discrete number and unit of measurement
☐	☒	A statement on whether measurements were taken from distinct samples or whether the same sample was measured repeatedly
☐	☒	The statistical test(s) used AND whether they are one- or two-sided<br>*Only common tests should be described solely by name; describe more complex techniques in the Methods section.*
☐	☒	A description of all covariates tested
☐	☒	A description of any assumptions or corrections, such as tests of normality and adjustment for multiple comparisons
☐	☒	A full description of the statistical parameters including central tendency (e.g. means) or other basic estimates (e.g. regression coefficient) AND variation (e.g. standard deviation) or associated estimates of uncertainty (e.g. confidence intervals)
☐	☒	For null hypothesis testing, the test statistic (e.g. F, t, r) with confidence intervals, effect sizes, degrees of freedom and P value noted<br>*Give P values as exact values whenever suitable.*
☒	☐	For Bayesian analysis, information on the choice of priors and Markov chain Monte Carlo settings
☐	☒	For hierarchical and complex designs, identification of the appropriate level for tests and full reporting of outcomes
☐	☒	Estimates of effect sizes (e.g. Cohen's *d*, Pearson's *r*), indicating how they were calculated

Our web collection on statistics for biologists contains articles on many of the points above.

Software and code

Policy information about availability of computer code

Data collection	No software was used for data collection
Data analysis	CellRanger v 6.0.1 was used to align reads to the GRCH38 reference and generate filtered count matrices. All other analyses were performed in R v4.2.1 and Python v3.10.12 using standard functions unless otherwise stated. Specific versions of packages used are listed in available GitHub code. The following packages were used: CellRanger v 6.0.1, singleCellTK v 2.8.0, Seurat v 4.3.0, scater v 1.24.0, harmony v 0.1.1, RColorBrewer v 1.1.3, ComplexHeatmap v 2.14.0, ArchR v 1.0.2, muscat v 1.12.1, readr v 2.1.4, ggplot2 v 3.4.2, ggsignif v 0.6.4, ggpubr v 0.6.0, magrittr v 2.0.3, scCoda v0.1.9 Python package. celda v1.19.1, hdWGCNA v0.4.5<br><br>HALO v3.6.4134.193,HALO AI v 3.6.4134, HALO Object Colocalization v2.1.4 algorithm and FISH v3.2.3 algorithm were used to analyze the histological and Nissl images. InForm v2.5.1 was used to spectrally unmix fluorescent in situ hybridization images.

For manuscripts utilizing custom algorithms or software that are central to the research but not yet described in published literature, software must be made available to editors and reviewers. We strongly encourage code deposition in a community repository (e.g. GitHub). See the Nature Portfolio guidelines for submitting code & software for further information.

Data

Policy information about availability of data

All manuscripts must include a data availability statement. This statement should provide the following information, where applicable:

- Accession codes, unique identifiers, or web links for publicly available datasets
- A description of any restrictions on data availability
- For clinical datasets or third party data, please ensure that the statement adheres to our policy

Data is available at GEO accession number GSE261807 . Code can be found at www.github.com/morganebutler/singleCellScripts. All other data supporting the findings of this study and unprocessed images are available upon reasonable request. Request for tissue or digital images can be made by emailing the corresponding author or through the Boston University ADRC request portal at https://wwwru.bumc.bu.edu/BUADC/RequestBrainTissue.aspx. The following public databases were used for the study: GRCH38 reference https://www.ncbi.nlm.nih.gov/datasets/genome/GCF_000001405.26/, Sun et al. https://compbio.mit.edu/microglia_states/, Visium spatial expression https://www.10xgenomics.com/datasets/adult-human-brain-1-cerebral-cortex-unknown-orientation-stains-anti-gfap-anti-nfh-1-standard-1-1-0. Sun and Akay et al. http://compbio.mit.edu/scADbbb/.

Research involving human participants, their data, or biological material

Policy information about studies with human participants or human data. See also policy information about sex, gender (identity/presentation), and sexual orientation and race, ethnicity and racism.

Reporting on sex and gender	CTE is a disease that is almost entirely reported in males, therefore the single nucleus RNA sequencing and RNA scope analysis was performed only on males. Due to the difficulty in obtaining controls, 5 females were included in the Nissl neuronal density quantification. No significant effect of sex was found. No information was collected on gender, therefore no gender-based analysis could be performed.
Reporting on race, ethnicity, or other socially relevant groupings	Race and ethnicity data were not available nor reported for participants in this study.
Population characteristics	Individuals included in this study were either healthy controls with no exposure to contact sports (n=8) or had a history of exposure to contact sports such as football (n=20). Within those exposed to repetitive head trauma through contact sports, 9 had a postmortem diagnosis of no CTE, and 11 had a postmortem diagnosis of Low CTE. Those included in the in situ hybridization analysis all had exposure to contact sports. Those included in the neuronal density analysis had a history of exposure to contact sports except for the healthy controls. All individuals were between the ages of 20 and 51 at death.
Recruitment	Participants were not specifically recruited for this study. Individuals were included from brain banks that collect tissue from voluntary donors.
Ethics oversight	Institutional review board approval for brain donation was obtained through the Boston University Alzheimer's Disease and CTE Center, National Center for PTSD, Human Subjects Institutional Review Board of the Boston University School of Medicine, VA Bedford Healthcare System, VA Boston Healthcare System, and the Iowa Neuropathology Resource Laboratory.

Note that full information on the approval of the study protocol must also be provided in the manuscript.

Field-specific reporting

Please select the one below that is the best fit for your research. If you are not sure, read the appropriate sections before making your selection.

☒ Life sciences ☐ Behavioural & social sciences ☐ Ecological, evolutionary & environmental sciences

For a reference copy of the document with all sections, see nature.com/documents/nr-reporting-summary-flat.pdf

Life sciences study design

All studies must disclose on these points even when the disclosure is negative.

Sample size	All available samples that met the criteria for inclusion and had tissue available at the time of the study were included in the single nucleus RNA sequencing, in situ hybridization, and neuronal density quantifications. No sample size calculation was performed. Sample sizes were determined based on querying our internal database and selecting all available samples that met our inclusion criteria outlined in the manuscript. The sample size was compared to other previous published single nucleus studies that found significant findings and was found to be consistent. Therefore, there was confidence in the sample size.
Data exclusions	35 samples were initially sequenced, 7 samples were excluded due to insufficient nuclei recovery during processing, poor quality of sequencing, or not meeting quality control standards such as gene count per cell, UMI counts etc.
Replication	Data was compared to other previously published datasets (Sun et al Cell 2023; Sun and Akay et al 2023) to demonstrate we could successfully replicate cell populations

Randomization	Samples were randomized within pathological group for processing of tissue into genomic libraries.
Blinding	Samples were not blinded for initial collection as the specific sample group and name was needed to identify and select the proper tissue from storage. Samples were blinded for tissue processing. For single nucleus RNA sequencing analysis, samples were blinded during tissue preparation and data QC. Samples were not blinded for comparative analyses as the pathologic grouping details were necessary for disease relevant comparisons. For immunohistochemistry, staining, and in situ hybridization experiments, observers were blinded when annotating , data collection, and analyzing tissue sections.

Reporting for specific materials, systems and methods

We require information from authors about some types of materials, experimental systems and methods used in many studies. Here, indicate whether each material, system or method listed is relevant to your study. If you are not sure if a list item applies to your research, read the appropriate section before selecting a response.

Materials & experimental systems

n/a	Involved in the study
☐	☒ Antibodies
☒	☐ Eukaryotic cell lines
☒	☐ Palaeontology and archaeology
☒	☐ Animals and other organisms
☒	☐ Clinical data
☒	☐ Dual use research of concern
☒	☐ Plants

Methods

n/a	Involved in the study
☒	☐ ChIP-seq
☐	☒ Flow cytometry
☒	☐ MRI-based neuroimaging

Antibodies

Antibodies used	Antibodies used: (Antibody, CAT#, Vendor, dilution, secondary species, antigen retrieval method).<br>SPP1, HPA027541, Sigma Aldrich, 1:500, Rb, AR9.<br>Iba1, 019-19741, Wako/Fujifilm, 1:500, Rb, AR6<br>AT8, MN1020, invitrogen, 1:500, M, AR6<br>Glut1, ab115730, abcam, 1:500, rb, AR9<br>P2Ry12, HPA013796, Sigma Aldrich, 1:2000, Rb, AR6
Validation	Antibodies were validated using positive control sections. Secondary antibodies were tested for non-specific binding to verify accuracy. Validation procedures are described on the manufactures website as listed below.<br>SPP1 https://www.sigmaaldrich.com/US/en/product/sigma/hpa027541<br>Iba1 https://www.fujifilmcdi.com/anti-iba1-polyclonal-antibody-019-19741<br>AT8 https://www.thermofisher.com/antibody/product/Phospho-Tau-Ser202-Thr205-Antibody-clone-AT8-Monoclonal/MN1020<br>GLUT 1 https://www.abcam.com/en-us/products/primary-antibodies/glucose-transporter-glut1-antibody-epr3915-ab115730<br>P2Ry12 https://www.sigmaaldrich.com/US/en/product/sigma/hpa013796

Plants

Seed stocks	*Report on the source of all seed stocks or other plant material used. If applicable, state the seed stock centre and catalogue number. If plant specimens were collected from the field, describe the collection location, date and sampling procedures.*
Novel plant genotypes	*Describe the methods by which all novel plant genotypes were produced. This includes those generated by transgenic approaches, gene editing, chemical/radiation-based mutagenesis and hybridization. For transgenic lines, describe the transformation method, the number of independent lines analyzed and the generation upon which experiments were performed. For gene-edited lines, describe the editor used, the endogenous sequence targeted for editing, the targeting guide RNA sequence (if applicable) and how the editor was applied.*
Authentication	*Describe any authentication procedures for each seed stock used or novel genotype generated. Describe any experiments used to assess the effect of a mutation and, where applicable, how potential secondary effects (e.g. second site T-DNA insertions, mosiacism, off-target gene editing) were examined.*

Flow Cytometry

Plots

Confirm that:

☒ The axis labels state the marker and fluorochrome used (e.g. CD4-FITC).

☒ The axis scales are clearly visible. Include numbers along axes only for bottom left plot of group (a 'group' is an analysis of identical markers).

☒ All plots are contour plots with outliers or pseudocolor plots.

☒ A numerical value for number of cells or percentage (with statistics) is provided.

Methodology

Sample preparation	Fresh frozen brain tissue was collected from the dorsolateral frontal cortex of each donor at the depth of the cortical sulcus. Visual delineation of grey/white matter was used to collect 50µg of tissue. Nuclei isolation and sorting were performed on two donor samples per day, randomizing for diagnosis and age. Tissue was kept on ice throughout nuclei isolation. Tissue was homogenized and lysed in NST Buffer with DAPI (146mM NaCl, 10mM Tris, 1mM CaCl2, 21mM MGCl2, 0.1%BSA, 0.1% NP-40, 40U/ml Protector RNase Inhibitor, DAPI) and snipped with scissors on ice for 10 minutes. Debris was removed using a 70µm filter. Cells were spun down and resuspended in nuclei storage buffer (2% BSA, 400U/mL Protector RNase Inhibitor) to reach a concentration of 500-1000 nuclei/µL. Nuclei were purified for DAPI positive cells with a FACS-Aria flow cytometer to remove debris.
Instrument	FACS-Aria
Software	No analysis was performed on flow cytometry data.
Cell population abundance	233,555 total cells were collected
Gating strategy	Gating was based on positive DAPI signal.

☒ Tick this box to confirm that a figure exemplifying the gating strategy is provided in the Supplementary Information.
